# Human leukocyte antigen (HLA) class I expression on Hodgkin–Reed–Sternberg cells is an EBV‐independent major determinant of microenvironment composition in classic Hodgkin lymphoma

**DOI:** 10.1002/hem3.84

**Published:** 2024-06-03

**Authors:** Berit Müller‐Meinhard, Nicole Seifert, Johanna Grund, Sarah Reinke, Fatih Yalcin, Helen Kaul, Sven Borchmann, Bastian von Tresckow, Peter Borchmann, Annette Plütschow, Julia Richter, Andreas Engert, Michael Altenbuchinger, Paul J. Bröckelmann, Wolfram Klapper

**Affiliations:** ^1^ Hematopathology Section and Lymph Node Registry, Department of Pathology University Hospital Schleswig‐Holstein Kiel Germany; ^2^ Department of Medical Bioinformatics University Medical Center Göttingen Göttingen Germany; ^3^ Department I of Internal Medicine, Center for Integrated Oncology Aachen Bonn Cologne Düsseldorf (CIO ABCD) and German Hodgkin Study Group (GHSG), Faculty of Medicine and University Hospital of Cologne University of Cologne Cologne Germany; ^4^ German Hodgkin Study Group (GHSG) Cologne Germany; ^5^ Department of Hematology and Stem Cell Transplantation, West German Cancer Center and German Cancer Consortium (DKTK partner site Essen), University Hospital Essen University of Duisburg‐Essen Essen Germany; ^6^ Mildred Scheel School of Oncology Aachen Bonn Cologne Düsseldorf (MSSO ABCD) Cologne Germany; ^7^ Max‐Planck Institute for Biology of Ageing Cologne Germany

## Abstract

Hodgkin–Reed–Sternberg cells (HRSCs) in classic Hodgkin Lymphoma (HL) frequently lack expression of human leukocyte antigen class I (HLA‐I), considered to hamper activation of cytotoxic T cells in the tumor microenvironment (TME). Here, we demonstrate HLA‐I expression on HRSCs to be a strong determinant of TME composition whereas expression of HLA‐II was associated with only minor differential gene expression in the TME. In HLA‐I‐positive HL the HRSC content and expression of CCL17/TARC in HRSCs are low, independent of the presence of Epstein–Barr virus in HRSCs. Additionally, HLA‐I‐positive HL shows a high content of CD8+ cytotoxic T cells. However, an increased expression of the inhibitory immune checkpoint LAG3 on CD8+ T cells in close proximity to HRSCs is observed. Suggesting interference with cytotoxic activity, we observed an absence of clonally expanded T cells in the TME. While HLA‐I‐positive HL is not associated with an unfavorable clinical course in our cohorts, they share features with the recently described H2 subtype of HL. Given the major differences in TME composition, immune checkpoint inhibitors may differ in their mechanism of action in HLA‐I‐positive compared to HLA‐I‐negative HL.

## INTRODUCTION

Classic Hodgkin Lymphoma (HL) is a B‐cell neoplasia most frequently involving young adults.^1^ Affected lymph nodes contain only scattered neoplastic so‐called Hodgkin and Reed–Sternberg cells (HRSCs) in an abundant nonneoplastic tumor microenvironment (TME), which is predominantly composed of CD4+ T cells and macrophages.^2^, ^3^ Immune cells in the TME are apparently ineffective in eliminating HRSCs for several reasons: Most T cells residing in the TME of HL express inhibitory immune checkpoint molecules, such as PD1, CTLA4, or LAG3.^4^, ^5^, ^6^, ^7^, ^8^ Moreover, HRSCs in HL are characterized by frequent loss of expression of human leukocyte antigen (HLA) proteins on the cell surface, suggesting that HRSCs may be undetectable for T cells.^9^ In the absence of HLA class I (HLA‐I) expression, cytotoxic CD8+ T cells are unlikely to recognize and attack HRSCs. Similarly, the absence of HLA class II (HLA‐II) on HRSCz hampers recognition, for example, by CD4+ T cells. Additionally, HRSCs have been described to secrete high levels of cytokines potentially attracting various immune cells into the TME of the affected lymph nodes.^10^ Accordingly, the vast majority of the TME in HL is considered to predominantly reflect a protective microenvironment rather than an actively ongoing immune response against the HRSCs.^11^, ^12^

Despite incomplete knowledge about the mechanisms, targeting the HL TME by immune checkpoint blockade (ICB) with anti‐PD1 antibodies has proven to be highly effective in primary and relapsed HL.^13^, ^14^ While single‐agent anti‐PD1 therapy is standard‐of‐care in relapsed or refractory HL, it rarely leads to cure in this setting.^15^, ^16^ Current therapeutic strategies in HL hence combine anti‐PD1 ICB with cytotoxic therapy in the first‐line setting and with other therapeutic modalities in the relapsed situation.^17^, ^18^ To fully leverage the potential of ICB in HL, understanding the interaction of HRSCs with T cells in the TME seems crucial. Recently published studies on the interplay between HL TME and ICB in HL have focused on HLA‐II since (i) HLA‐II is much more frequently found on HRSCs compared to HLA‐I,^9^ (ii) expression of HLA‐II was associated with favorable response to ICB in refractory/relapsed HL,^15^, ^19^ and (iii) HLA‐II expression was shown to be associated with a distinct spatial arrangement of the TME.^6^ In contrast, the effects of HLA‐I expression in HL remain poorly understood.

Assuming that an intact expression of HLA molecules on the surface of HRSCs is a prerequisite for an effective cytotoxic T‐cell immune response directed against the neoplastic cells, we herein investigate the immune landscape of HL in relation to the expression of HLA molecules on HRSCs. Albeit detectable only in a relatively small subgroup of HL, HLA‐I expression on HRSCs was associated with a distinct gene expression profile of a specific cellular composition of the TME but not with clonal expansion of T cells.

## MATERIALS AND METHODS

### Patients and tissue

Gene expression profiling (*n* = 95), T‐cell receptor (TCR) repertoires (*n* = 90), and whole slide image (WSI) analysis (*n* = 106) were performed on primary HL specimens from patients enrolled in the prospective investigator‐initiated German Hodgkin Study Group (GHSG) randomized multicenter phase II NIVAHL trial (NCT03004833). In NIVAHL, adult early‐stage unfavorable HL patients were randomized to receive either four double cycles of concomitant treatment with the anti‐PD1 antibody nivolumab and AVD (4×N‐AVD, group A) or sequential 4×nivolumab, 2×N‐AVD, and 2×AVD (group B), each followed by 30 Gy involved‐site radiotherapy in all patients.^20^ The primary analysis of the NIVAHL clinical trial with details regarding its methodology, patient characteristics, safety, and efficacy including the currently available 1‐year follow‐up has been described,^20^ and recently the final analysis confirmed excellent long‐term outcomes.^14^ All patients reported herein provided informed consent for the respective translational analyses.

Due to the excellent outcomes, the NIVAHL trial is unsuitable for the analysis of prognostic parameters for progression‐free survival (PFS). Thus, we additionally analyzed HLA expression on HRSCs in a cohort of advanced‐stage HL treated with conventional chemotherapy (BEACOPP‐based) in the GHSG phase III trials HD12^21^ and HD15,^22^ for which gene expression and WSI analysis data have already been published.^3^, ^23^ Details on these cohorts including their unbiased nature with respect to the whole study cohort of the clinical trials have been described previously.^3^, ^23^

Additional patients to be analyzed by multistaining immunofluorescence (*n* = 20) and TARC‐CD30 double staining (*n* = 20) were selected from a cohort of patient specimens based on the HLA expression status and availability of sufficiently large tissue biopsies (Supporting Information S1: Table 3). Analyses were conducted according to the recommendations of the ethics advisory committee of the Medical Faculty of the University of Kiel (D464/17).

### HLA expression

Expression of HLA proteins was measured according to published protocols.^9^ Briefly, HLA‐I was analyzed by β‐2 microglobulin (b2M) expression and HLA‐II by HLA‐DP/DQ/DR expression using conventional immunohistochemistry (IHC; Supporting Information S1: Table 1). HRSCs were identified by experienced observers and HLA expression was scored positive if more than 50% of HRSCs showed unambiguous staining of the membrane following the procedure of previous publications.^9^, ^24^

### Gene expression profiling

DNA and RNA extraction was performed using different commercial kits (Qiagen, AmpTec, or Thermo Fisher Scientific) according to the manufacturer's instructions. Gene expression analysis was performed using the NanoString PanCancer Immune Profiling Panel (NIVAHL cohort)^19^ or a custom panel as previously described.^23^, ^25^ Background thresholding and normalization were performed by the NSolver software (version 4.0; NanoString Technologies). The R package “nanostring” was used to perform quality controls.^26^ Samples with ≤50% of genes detected above the limit of detection were removed (*n* = 2). Additionally, for analysis of differential gene expression, two samples were removed, because of the nonevaluable HLA status of HRSCs by IHC. Gene expression counts of the remaining samples (*n* = 91) were log 2‐transformed and subsequently centered around the sample mean. Gene expression data of *n* = 8 primary biopsies reanalyzed herein were part of a previous publication.^11^

### Analysis of differential gene expression

An univariate generalized linear model was used to statistically compare the gene expression between HLA‐I/‐II‐positive (HLA‐I/‐II+) and HLA‐I/‐II‐negative (HLA‐I/‐II−) samples, where HLA‐I/‐II status served as the response and each gene as a predictor variable. To take a possible effect of EBV on HLA‐I/‐II status into account, an additional analysis was performed, where the EBV status of each patient assessed by EBV‐encoded RNA in situ hybridization or LMP1 IHC was added to the generalized linear model function as a second predictor variable. The fold‐change for each gene was calculated by subtracting the mean (log) HLA‐I/‐II+ gene expression from the mean (log) HLA‐I/II− gene expression. All genes with a *p* value ≤0.05 and a fold‐change >0.6 (or ≤−0.6) were considered significantly upregulated (or downregulated) in HLA‐I/I− samples in volcano plots. All *p* values were also adjusted for multiple testing using the Benjamini–Hochberg procedure.

### Immunofluorescence multistaining and WSI analysis

WSI analysis of conventional staining for CD8, CD20, and CD30 was performed of full tissue section using TissueStudio 64 (Definiens) as previously described.^3^ Multiplex immunofluorescence staining was performed using Opal 6‐Plex Manual Detection Kit—for Whole Slide Imaging (Akoya Biosciences). The staining consisted of a panel that included CD30 to detect HRSC, CD68, LAG3, FoxP3, and CD8, and a second panel, which included CD30, CD3, PD1, and CD8 or a double staining of CD30 and CCL17/TARC (Supporting Information S1: Table 1). Digital images were captured from fluorescence scans and analyzed by QuPath software.^27^

### TCR repertoire

TCR receptor analysis of bulk tissue was performed for the β‐chain of the TCR using the service of Adaptive Biotechnologies (https://www.adaptivebiotech.com) according to the manufacturer's guidelines in 90 samples.

### Statistical analysis

Statistical analysis was performed using GraphPad Prism 7 for Windows (version 7.02) with comparisons of groups by paired or unpaired *t*‐test.

## RESULTS

### HLA expression in HRSCs of HL patients treated in the NIVAHL trial

**Figure 1:**
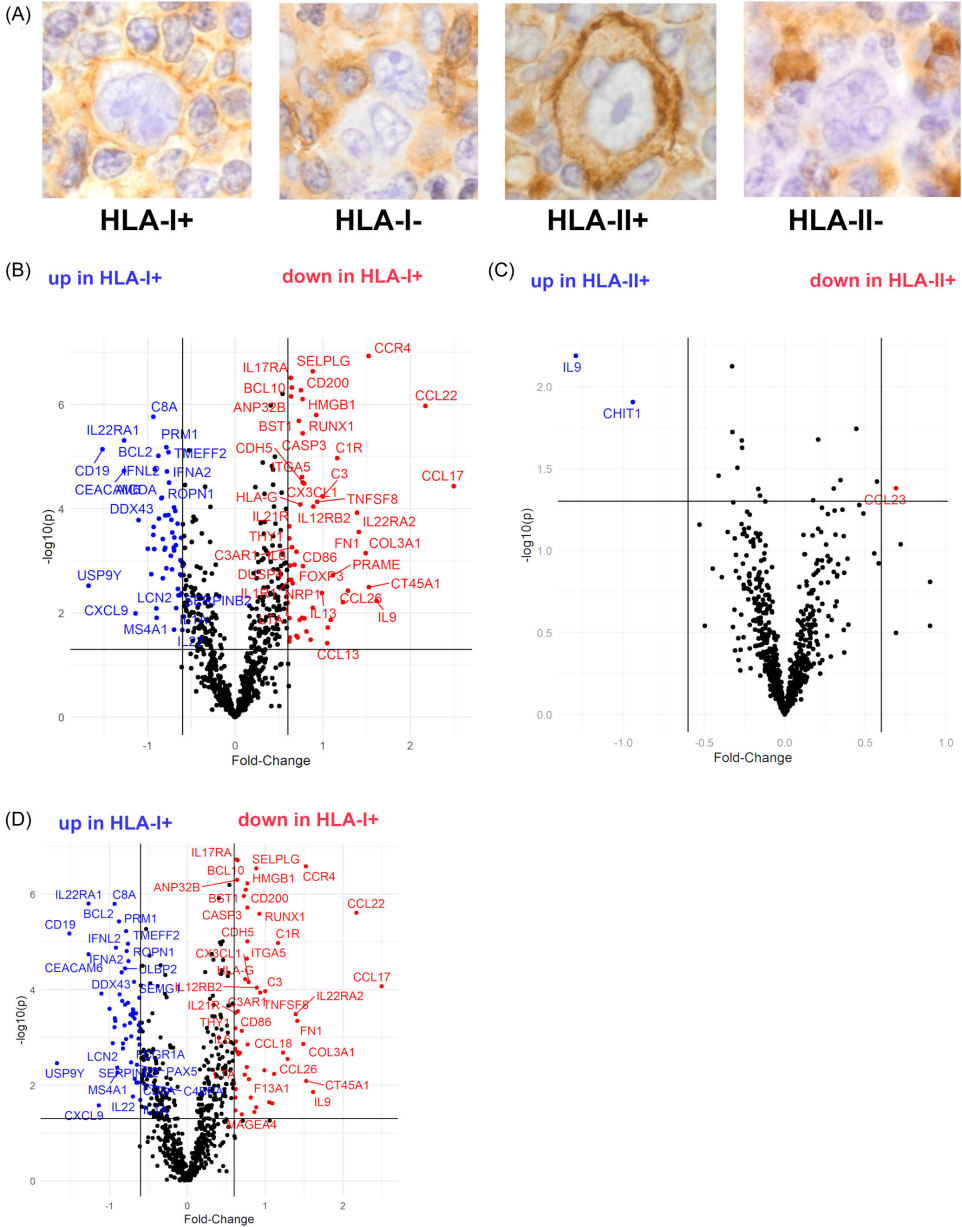
(A) Representative examples of Hodgkin–Reed–Sternberg cells (HRSCs) human leukocyte antigen‐1‐positive (HLA‐I+), HLA‐I−, HLA‐II+, and HLA‐II− (left to right). (B) Volcano plot comparing bulk gene expression of Hodgkin lymphoma (HL) stratified according to HLA‐I expression on HRSCs (total *n* = 97 with HLA‐I information, *n* = 11 HLA‐I+ cases). (C) Volcano plot comparing bulk gene expression of HL stratified according to HLA‐II expression on HRSCs (total *n* = 98 with HLA‐II information, *n* = 53 HLA‐II+ cases). (D) Volcano plot comparing bulk gene expression of HL stratified according to HLA‐I expression on HRSCs with Epstein–Barr virus status of HRSCs as a confounder (total *n* = 97 with HLA‐I information, *n* = 11 HLA‐I+ cases).

In line with previous reports, expression of HLA was frequently lost on HRSCs (Figure 1A): HLA‐I was positive in 11/97 (11%) and HLA‐II was positive in 53/98 (54%) of NIVAHL cases with an evaluable result. Key features of cases with and without HLA‐I+ HRSCs in the NIVAHL cohort are summarized in Table 1. Co‐expression of HLA‐II was not significantly different in HLA‐I+ versus HLA‐I− cases (Table 1). HLA‐I+ HL was more frequently of mixed cellularity subtype compared to HLA‐I− cases (3/11, 27% and 12/86, 14%, respectively; Table 1). The histology subtype nodular sclerosis was less frequent in HLA‐I+ compared to HLA‐I− HL (3/11, 27% and 66/86, 77%, respectively, *p* = 0.0004 for distribution of histology subtypes; Table 1). As reported previously,^28^ HLA‐I+ HRSCs showed significant enrichment for EBV‐positivity when compared to HLA‐I− HL (5/11 (45%) and 7/86 (8%), respectively, *p* = 0.0038). Details on the clinical features of patients with HLA‐I+ and HLA‐I− HL are shown in Supporting Information S1: Table 2.

**Table 1:** HLA expression, EBV status, and histologic HL subtype of patients treated in the NIVAHL trial.

NIVAHL	HLA‐I+ (11/105, 10%)	HLA‐I− (86/105, 82%)	HLA‐I−na (8/105, 8%)	*p* Value
HLA status				
HLA‐II+	7/11 (64%)	46/86 (54%)	0/8 (0%)	0.7494^a^
HLA‐II−	4/11 (36%)	39/86 (45%)	2/8 (25%)	
HLA‐II‐na	0/11 (0%)	1/86 (1%)	6/8 (75%)	na
HL subtype				
Nodular sclerosis	3/11 (27%)	66/86 (77%)	1/8 (13%)	0.002^b^
Mixed cellularity	3/11 (27%)	12/86 (14%)	0/8 (0%)	
Others	2/11 (18%)	1/86 (15%)	0/8 (0%)	
na	3/11 (27%)	7/86 (8%)	7/8 (88%)	na
EBV status				
EBV+	5/11 (45%)	7/86 (8%)	2/8 (25%)	0.0058^a^

Abbreviations: EBV, Epstein–Barr virus; HL, Hodgkin lymphoma; HLA, human leukocyte antigen; na, not analyzed.

^a^ Fisher's exact test, statistical test calculated for HLA‐I+ versus HLA‐I−.

^b^ *χ* ^2^ test, statistical test calculated for HLA‐I+ versus HLA‐I−.

### HLA‐I expression is associated with the TME composition independent of EBV

**Figure 2:**
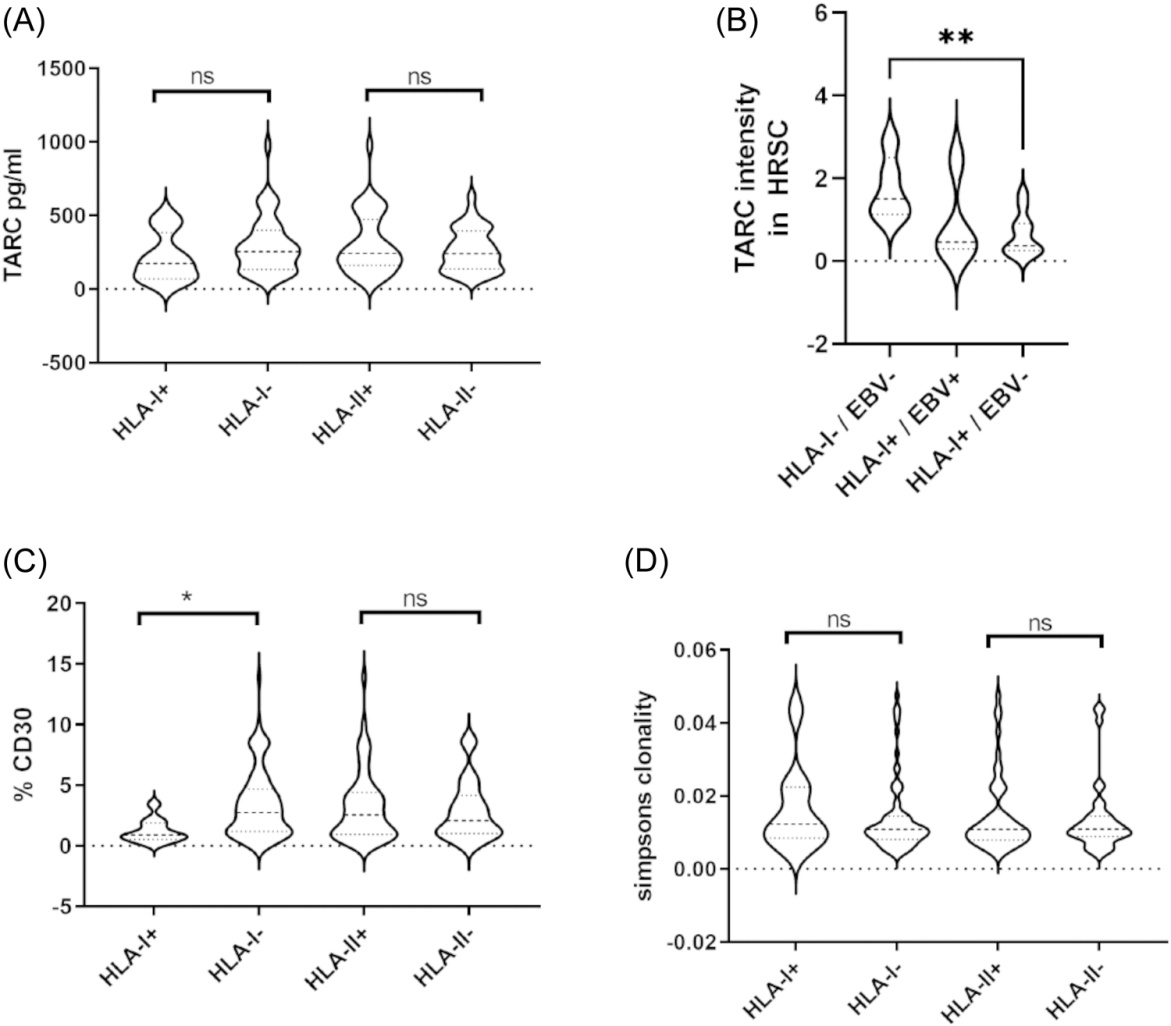
(A) Levels of TARC/CCL17 in the serum of patients. (B) Quantitative expression of TARC/CCL17 protein in Hodgkin–Reed–Sternberg cells (HRSCs) by image analysis of TARC/CD30 double staining. (C) Percent of the area covered by CD30+ HRSCs assessed by whole slide image analysis. (D) T‐cell receptor repertoire analysis showing Simpson's clonality. HLA, human leukocyte antigen; ns, not significant. *p < 0.05 and **p < 0.01.

To investigate associations of HLA expression on HRSCs with features of the TME, we determined differentially expressed genes by bulk gene expression via the NanoString technique. We observed relevant differences in TME gene expression when cases were divided according to the absence or presence of HLA‐I on HRSCs (Figure 1B and Supporting Information S1: Table 3). In contrast, HLA‐II expression on HRSCs had little effect on differential gene expression of the TME (Figure 1C). Since HLA‐I expression is frequently associated with the presence of EBV in HRSCs, we calculated differential gene expression using EBV as a confounding variable yielding very similar results (Figure 1D). Moreover, we analyzed previously published gene expression data composed of a restricted set of genes (*n* = 142) in an independent cohort of advanced‐stage HL.^23^ Again, HLA‐I expression of HRSCs was associated with reduced expression of CCL17/TARC and CCL22 independently of the presence of EBV (Supporting Information S1: Figure 1). Of Note, the effect of downregulated CCL17/TARC and CCL22 was even detectable when the analysis was restricted to EBV+ lymphomas (Supporting Information S1: Figure 2). These data suggest that HLA‐I outweighs HLA‐II expression as a determinator of TME composition in HL and that the effect of HLA‐I expression on the gene expression status is independent of the EBV status of HRSC.

### Low expression of CCL17/TARC and low HRSC content in HLA‐I+ HL independent of EBV

Key cytokines derived from HRSC (e.g., CCL17/TARC, CCL22, IL9, IL13) were significantly lowly expressed in HL with HLA‐I+ compared to cases with HLA‐I− HRSCs (Supporting Information S1: Table 3). Moreover, cancer testis antigens and TNFRSF8 (CD30; Supporting Information S1: Figures 1 and 2) were among the downregulated genes potentially reflecting reduced HRSC content. Thus, we evaluated if the differences in gene expression according to HLA‐I status depend on the capacity of HRSCs to secrete cytokines or if they are simply attributed to differences in the abundance of HRSCs in the tissue. First, we evaluated serum levels of TARC/CCL17 while treating naïve patients according to the HLA expression status of HRSCs but did not detect any significant difference (Figure 2A). Of note, TARC in the tissue was analyzed at the RNA level and not as protein, which may explain the lack of correlation. To understand if low TARC/CCL17 levels are derived from lower expression within HRSCs, we evaluated TARC/CCL17 protein expression quantitatively in HRSCs using immunofluorescence double staining of CD30 and TARC a method that is independent of HRSC quantity. In fact, TARC/CCL17 protein expression was found to be significantly lower in HLA‐I+ compared to HLA‐I− cases when the analysis was restricted to EBV− lymphomas (Figure 2B). HL with EBV+ HRSCs shows an intermediate TARC/CCL17 protein expression, which was not significantly different from HLA‐I+/EBV− cases, indicating that both EBV and HLA‐I expression influence TARC/CCL17 expression of HRSCs but HLA‐I expression is the major factor to be associated with downregulation of TARC/CCL17 on the protein level.

Gene expression showed significantly lower levels of cancer testis antigens (CT45, PRAME) in HLA‐I+ compared to HLA‐I− cases (Supporting Information S1: Table 3) and CD30 (Supporting Information S1: Figures 1 and 2). Thus, we evaluated HRSC content in the tissue using WSI analysis of CD30‐stained full slides as previously described^3^ to assess the content of tumor cells in the tissue as representative as possible. By WSI analysis, the relative content of HRSCs was significantly lower in HLA‐I+ compared to HLA‐I− HL (Figure 1C). When the cases were sorted according to HLA‐II expression on HRSCs, no significant difference in HRCS content was observed (Figure 2C).

**Figure 3:**
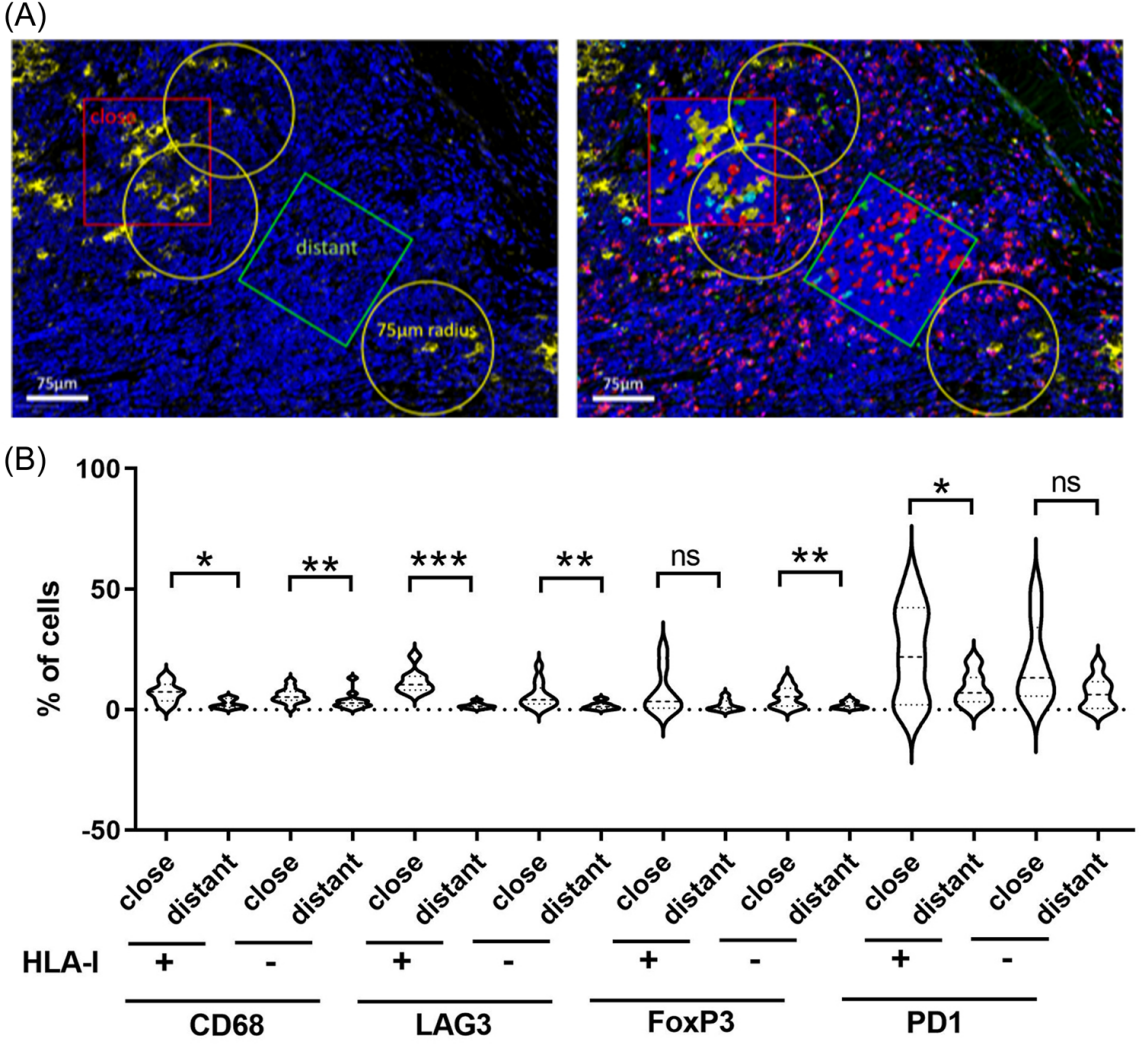
(A) Spatial analysis strategy of immunofluorescence multistaining. Hodgkin–Reed–Sternberg cells (HRSCs) stained yellow in the left panel illustrate the selection procedure of areas. Areas are depicted as follows. Red box: Area containing HRSCs (“close”). Yellow circles: 75 µm radius around scattered HRSCs. Green box: Microenvironment with a distance of at least 75 µm from neighboring HRSCs (“distant”). Right panel includes all cell types detected by the staining panel with CD30 (yellow), CD68 (green), LAG3 (turquoise), FoxP3 (pink), and CD8 (red) and corresponding cell detections conducted by QuPath. (B) Percent of cells in the respective areas as shown above according to human leukocyte antigen‐I expression status. ns, Not significant. *p < 0.05, **p < 0.01, and ***p < 0.001.

We conclude that expression of cytokines associated with HRSCs is low in the tissue of HLA‐I+ cases both due to low expression levels in HRSCs and also due to low content of HRSC. Of note, pathological inspection revealed a pattern of complete infiltration of the lymph nodes by HL in the vast majority of cases independent of the HLA expression status. Consequently, cases with HLA‐I+ HRSCs did not cluster together or more closely to reactive lymph nodes in a principal component analysis based on NanoString gene expression (Supporting Information S1: Figure 3). Thus, partial infiltration of tissue does not explain low levels of HRSCs in HLA‐I+ cases. Instead, the group of HL with HLA‐I+ HRSCs seems to be characterized by a low tumor cell content and HRSCs producing lower levels of HL‐typical cytokines. Of note, CCR4, the receptor of TARC/CCL17 and CCL21, was also among the genes significantly downregulated in HLA‐I+ lymphomas.

### HLA‐I expression of HRSCs is associated with increased cytotoxic T‐cell content but not with TCR clonality

**Figure 4:**
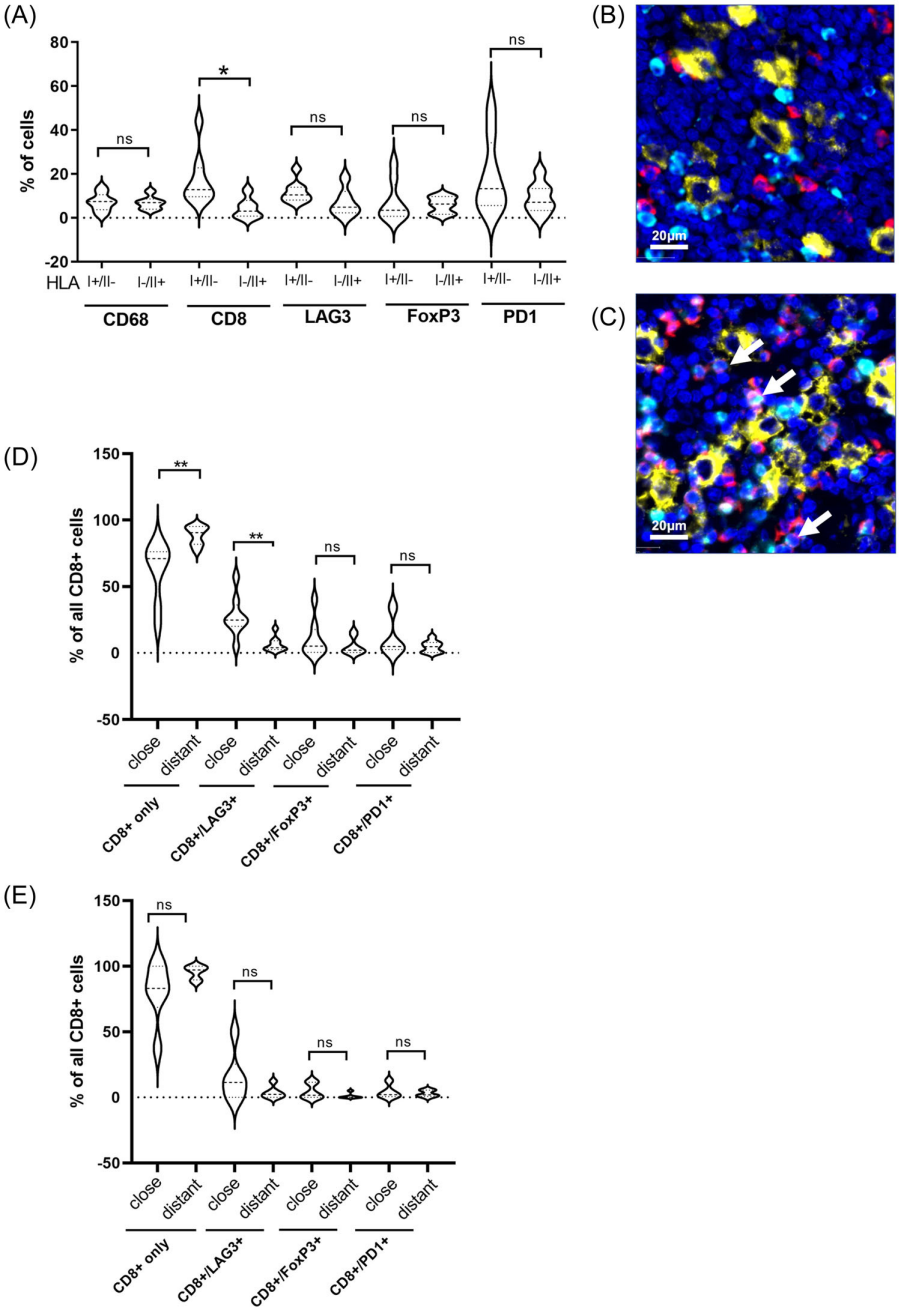
(A) Analysis of areas in close proximity to Hodgkin–Reed–Sternberg cells (HRSCs) restricted to cases expressing human leukocyte antigen‐I (HLA‐I) but not HLA‐II (HLA‐I+/II−) or HLA‐II but not HLA‐I (HLA‐I−/II+). Percent of cells in the respective areas indicated. ns, Not significant. *p < 0.05. (B and C) Immunofluorescence multistaining of a case with HLA‐I− (B) and a case with HLA‐I+ HRSCs (C). CD30 (yellow), CD8 (red), and LAG3 (turquoise). Arrows in (C) indicate CD8+ cells co‐expressing LAG3. (D and E) Immunophenotype of CD8+ cells as CD8+ lacking LAG3, FoxP3, and PD1 (CD8+ only) or co‐expressing the respective marker. Percentage of cells with phenotype relative to all CD8+ cells is shown. (D) HLA‐I+ lacking HLA‐II expression and (E) cases with HLA‐II+ HRSCs lacking HLA‐I expression. n.s., Not significant. *p < 0.05 and **p < 0.01.

The presence of HLA‐I on HRSCs was associated with a significant upregulation of cytotoxicity associated gene (e.g., CD8B) compared to HLA‐I− cases (Supporting Information S1: Table 3). To confirm that this gene expression difference reflects cellular composition, we performed CD8 IHC and WSI analysis to quantify all CD8 cells within the TME. HLA‐I expression of HRSCs was in fact associated with a higher content of CD8+ T cells (Supporting Information S1: Figure 4). To understand if the cytotoxic immune response is associated with clonal expansion of T cells, TCR repertoires analyzed in bulk tissue were compared between HL with HLA‐I+ and HLA‐I− HRSCs. As previously published for a much smaller cohort, the TME of HL was highly polyclonal when compared to solid tumors such as breast cancer.^11^ However, we did not detect increased clonality in HL with HLA‐I+ HRSCs (Figure 2D), indicating mechanisms preventing the activation and clonal expansion of cytotoxic T cells in HLA‐I+ HL.

### Spatial accumulation of CD8+/LAG3+ cells near HLA‐I+ HRSC

The TME in HL has been shown to display spatial arrangements that depend on HLA expression on HRSC. Using multicolor immunofluorescence staining, we separated areas close to HRSCs (defined as areas with a maximum of 28.33 µm away from HRSCs) and areas distant to HRSCs (>75 µm distant to HRSCs; Figure 3A) in cases with and without HLA‐I expression on HRSCs. CD68+ macrophages, LAG3+, FoxP3, and PD1+ T cells were enriched in close proximity or HRSCs irrespective of HLA‐I status, but this effect was not statistically significant for FoxP3 and PD1 in HLA‐I− cases (Figure 3B).

Given the discrepancy between signatures of a cytotoxic immune response detectable in global gene expression and WSI analysis on one hand and the absence of clonal expansion of T cells on the other, we analyzed if spatial arrangements interfere with a cytotoxic immune response of CD8+ cells in HL expressing HLA‐I. Restricting the analysis to the immediate proximity to HRSCs and cases that either express HLA‐I but not HLA‐II or HLA‐II but not HLA‐I indicates that the accumulation of CD8+ cells in close proximity to HRSCs is linked to the HLA expression status (Figure 4A). Importantly, the spatial arrangement of CD8 T cells was also found when EBV+ cases were excluded (Supporting Information S1: Figure 5). Thus, the presence of HLA‐I on HRSCs is associated with a global increase in signatures of a cytotoxic immune response as well as local accumulation of CD8+ cytotoxic T cells in close proximity to HRSCs and this effect is independent of the presence of EBV in HRSCs.

To understand what additional features might prevent clonal expansion and activation of such T cells we determined the phenotype of CD8+ T cells with respect to the expression of PD1 and LAG3, an inhibitory molecule found to be abundantly present in the microenvironment of HL, and expression of FoxP3 as a marker for regulatory T cells (Figure 4B+C). CD8+ cells lacking LAG3 and FoxP3 (CD8 only) were found to decrease close to HRSCs, whereas CD8+ cells co‐expressing either LAG3, FoxP3, or PD1 were enriched close to HRSCs compared to more distant areas but the effect was only significant for LAG3 (Figure 4D). TIM3, expression on CD8 cells was enriched in HLA‐I+ cases but no spatial difference between cells close or distant to HRSCs was detected (Supporting Information S1: Figure 6). Thus, the immediate proximity of HLA‐I+ HRSCs is enriched for CD8+ cytotoxic cells but these cells are characterized by LAG3 expression, potentially interfering with their ability for activation and clonal expansion. The spatial nature of the phenotype described suggests that HLA‐I+ HRSCs might create a milieu in their proximity preventing CD8+ cell activation by inducing LAG3 on CD8+ cells. This phenomenon was not detectable at a significant level when cases were stratified according to HLA‐II expression status, although a similar trend was detectable (Figure 3E).

### HLA expression and outcome

Reports on the clinical relevance of HLA expression on HRSCs in HL after conventional polychemotherapy are contradictory with the major studies published showing either unfavorable outcome of HLA‐I+^29^ or HLA‐II+ cases,^30^ whereas others did not find an association of HLA on HRSCs with outcome after conventional chemotherapy.^31^ Since the excellent outcome in the NIVAHL trial prohibits analyses toward PFS, we reanalyzed a previously published cohort of patients with advanced‐stage HL treated in the GHSG HD12 and HD15 trials. We have previously shown that B‐cell content assessed by WSI analysis is associated with a more favorable outcome of HL.^3^, ^32^ Of note, several genes reflecting B cells (CD20/MS4A1, CD19, CD22, CD79B) were significantly highly expressed in HLA‐I+ compared to HLA‐I− lymphomas (Supporting Information S1: Table 2), suggesting that the prognostically favorable B‐cell‐rich TME is a feature of HLA‐I+ HL. In the joint HD12/HD15 cohorts, HLA‐I+ HL showed a trend toward favorable PFS without reaching significance (supplementary figure 7). However, while analyzing the trials separately, significance for PFS was reached in the HD15 trial (Supporting Information S1: Figure 8).

## DISCUSSION

In the vast majority of HL, HLA‐I expression is lost on HRSCs due to inactivating mutations of b2M.^33^, ^34^ However, a subset of HL may retain HLA‐I expression. Since HLA‐I expression is associated with the histology subtype of mixed cellularity and EBV‐positivity of HRSC, its proportion varies in relation to the baseline characteristics of the study cohort. In adult patients from central Europe like Germany nodular sclerosis is the predominant histology pattern of HL and thus the proportion of HLA‐I+ HL is low (11% and 17% in the two cohorts of the NIVAHL and HD12/15 trials studied herein, respectively). However, in other studies including younger patients and in patient cohorts from geographical regions outside of Europe, the group of HLA‐I+ HL may be higher.^9^, ^35^, ^36^, ^37^ While the subgroup of HLA‐I+ HL may be small, it appears to be distinct in its TME composition and has potential therapeutic implications. In fact, a cytotoxic immune response with increased CD8+ T cells in the TME of HLA‐I+ HL has been suggested previously.^35^ Our data confirm this observation but add relevant additional information and context: HL with preserved HLA‐I expression contains less tumor cells and these express lower levels of HRSC‐associated cytokines. Thus, HLA‐I+ HL may be less competent to remodel their microenvironment in a typical HL‐specific manner. Additionally, the presence or absence of HLA‐I on HRSCs is a major determinant of bulk gene expression linking biological features of HRSCs and the TME, respectively. It is important to stress, that herein the association of HLA‐I expression on HRSCs with TME composition was independent of the presence of EBV. Our data corroborate the concept that features of HRSCs other than the presence of EBV are to be linked to the composition of the TME.^38^

The most relevant finding of our study is the lower tumor cell content and the low levels of CCL17/TARC expression in HLA‐I+ HL. CCL17/TARC has been reported to be a marker of poor prognosis when detected in its soluble form in the serum or by RNA expression in the TME.^39^ Of note, in addition to CCL17/TARC other genes overexpressed in HLA‐I+ HL have previously been shown to be associated with HL prognosis, such as CD30 and PDGFRA.^23^ Thus, the HLA‐I+ phenotype of HRSCs associates with a gene expression signature of favorable prognosis. Nevertheless, HLA‐I expression by IHC may not be a sufficient surrogate biomarker for this favorable subgroup as we do not detect the association of HLA‐I expression with prognosis in the cohorts studied herein. Nevertheless, we can exclude that HLA‐I is associated with inferior prognosis as previously published.^29^ Instead, we found an association of HLA‐I expression with a B‐cell‐rich microenvironment that has repeatedly been shown to be associated with a favorable prognosis.^3^, ^32^ Since CCL17/TARC is considered a key cytokine to shape the TME in HL, the low expression in HLA‐I+ HL appears to directly influence TME composition in this subtype.^10^

The efficacy of ICB such as anti‐PD1 in solid cancers has been linked to CD8+ cytotoxic T‐cell activation in the TME of these tumors.^40^ The absence of HLA‐I on HRSCs raised doubts about a similar mechanism of action in HL. Instead, the very favorable effects of ICB in HL have largely been attributed to the expression of HLA‐II and thus to activation of CD4+ T cells as well as to early T‐cell independent effects on the TME such as reverse signaling or T‐cell activity without proliferation.^5^, ^6^, ^11^, ^19^, ^41^ Our present study suggests that the small but biologically distinct subgroup of HLA‐I+ HL elicits a cytotoxic immune response that is probably blocked by induction of inhibitory checkpoint molecules on CD8+ T cells in close proximity to HRSCs like LAG3. Thus, ICB in HLA‐I+ HL may benefit from combined ICB or application schemes allowing cytotoxic T cells to expand before the addition of cytotoxic drugs, which potentially inhibit T‐cell proliferation. The data presented in the current study point toward the potential for a cytotoxic immune response in HLA‐I+ but not HLA‐I− HL. This is underscored by the content of CD8+ cells and their spatial arrangement but interestingly not reflected by clonal TCR expansions in bulk tissue analysis. Whether a subtle clonal expansion of T cells occurs in HLA‐I+ HL cannot be answered by the analysis in our study and requires single‐cell analysis of HL tissues with known HLA expression status, ideally during anti‐PD1 treatment. Unfortunately, these biopsies are rare and the material is often sparse, thus hampering a more detailed analysis of clonal expansion of cytotoxic T cells in this specific subtype of HL.^11^ Moreover, it is currently impossible to evaluate the prognostic impact of HLA‐I expression on HRSCs when patients are treated with ICB in the first line since relapses in this patient cohort are exceptionally rare so far.^14^, ^20^

Our data are in line with the concept of genetic subtypes of HL as recently published.^38^ The H2 subtype of HL described by Alig et al. shares features of our subgroup of HLA‐I+ HL like the presence of EBV, high level of CD8+ T cells, and low expression of CCL17/TARC. Additionally, this subtype is assumed to show preserved HLA expression, although HLA expression was not evaluated directly by IHC.^38^ It should be noted that assessing HLA expression by IHC requires sufficiently large tissue biopsies of appropriate quality and experienced observers since analysis by visual inspection remains a challenge. Several questions need to be addressed before HLA expression can be applied as a clinical biomarker, including the cut‐off for positivity,^9^ interobserver variability, and the optimal method of staining and evaluation. Finally, it will be important to assess the overlap of the H2 subtype of HL with the group of HLA‐I+ HL by IHC in cohorts for which both technologies have been applied.

The clinical significance of HLA‐I expression of HRSCs remains currently uncertain and requires further studies to understand in which patient subgroups HLA‐I expression is a prognostic factor under polychemotherapy. We do not envision a causal connection between HLA‐I expression on HRSC and effects of conventional chemotherapy. Thus, lack of prognostic relevance of HLA‐I expression in trials using polychemotherapy does not make our findings less relevant. Given the significant association of HLA‐I expression with microenvironmental composition this subtype likely will be therapeutically and prognostically relevant only under ICB. Testing this hypothesis requires clinical trials including ICB in which follow‐up and events are sufficient to perform prognostic analysis. To the best of our knowledge, these trials are currently still ongoing. Future studies should include quantitative spatial analysis, e.g. for LAG3 on CD8+ since such analysis have recently identified clinically relevant subgroups that are not detectable by bulk analysis conducted in our study.^42^

Our data indicate that a systematic assessment of HLA expression on HRSCs and a more detailed characterization of the HLA‐I+ subgroup require specific attention in future analyses. This approach may help to understand the mechanism of action of anti‐PD1 in HL and thus optimize clinical use of ICB across different treatment scenarios.

## AUTHOR CONTRIBUTIONS

Sarah Reinke, Michael Altenbuchinger, and Wolfram Klapper conceived and designed the overall studies. Paul J. Bröckelmann and Andreas Engert led the clinical trials. Berit Müller‐Meinhard, Johanna Grund, Sarah Reinke, Nicole Seifert, Paul J. Bröckelmann, Fatih Yalcin, Helen Kaul, and Annette Plütschow generated and analyzed data. Julia Richter, Bastian von Tresckow, Peter Borchmann, and Andreas Engert provided patient data and material. Sarah Reinke and Wolfram Klapper wrote the manuscript. All authors revised the manuscript and agreed with its final version.

## CONFLICT OF INTEREST STATEMENT

Dr von Tresckow is an advisor or consultant for Allogene, BMS/Celgene, Cerus, Incyte, IQVIA, Gilead Kite, Lilly, Miltenyi, Novartis, Noscendo, Pentixapharm, Roche, Amgen, Pfizer, Takeda, Merck Sharp & Dohme, and Gilead Kite; has received honoraria from AstraZeneca, BMS, Incyte, Lilly, Novartis, Roche Pharma AG, Takeda, and Merck Sharp & Dohme; reports research funding from Novartis (Inst), Merck Sharp & Dohme (Inst), and Takeda (Inst); and reports travel support from AbbVie, AstraZeneca, Gilead Kite, Lilly, Merck Sharp & Dohme, Pierre Fabre, Roche, Takeda, and Novartis all outside the submitted work. Dr P. Borchmann reports grants from BMS during the conduct of the study. Dr Engert reports grants and nonfinancial support from BMS during the conduct of the study, and personal fees from Takeda, BMS, and MSD outside the submitted work. Dr Bröckelmann is an advisor or consultant for BeiGene, BMS, MSD, Stemline, and Takeda, received honoraria from BeiGene, BMS, MSD, Stemline, and Takeda, received travel support from BeiGene, Celgene, and Takeda, and reports research funding from BeiGene (Inst), BMS (Inst), MSD (Inst) and Takeda (Inst). Dr Klapper reports grants from Roche, Amgen, Takeda, Incyte, and Regeneron paid to his institution outside the submitted work. The remaining authors declare no conflicts of interest.

## FUNDING

The authors thank Johanna Veldman and Arjan Diepstra for their help with scoring HLA staining. This work was supported by a grant from the German Cancer Aid (Deutsche Krebshilfe No. 70112502). Bristol Myers Squibb financially supported the NIVAHL trial. Scanning and image analysis technology was supported by the Kinderkrebsinitiative Buchholz, Holm‐Seppensen (KKI). TCR repertoire analysis was supported by a Young Investigator Award of Adaptive Biotechnologies April 2021 for Johanna Grund.

## Supporting information

Supporting information.

## Data Availability

The data that support the findings of this study are available from the corresponding author upon reasonable request. Pseudonymized original data will be made available upon reasonable request to the corresponding author: wolfram.klapper@uksh.de. TCR data are stored at Adaptive Biotechnologies.
