# Supplementary material for: Human leukocyte antigen (HLA) class I expression on Hodgkin–Reed–Sternberg cells is an EBV‐independent major determinant of microenvironment composition in classic Hodgkin lymphoma
Source: Hemasphere. 2024 Jun 3;8(6):e84. doi: 10.1002/hem3.84 (PMC11145947; doi:10.1002/hem3.84)
Supplement: Supplementary file 1 — Supporting information. [file HEM3-8-e84-s001.docx]

Supplementary data

**Fluorescence multi-staining and image analysis**

From the lymph node tissue samples fixed in formalin and embedded in paraffin, 2-3µm thick tissue sections were prepared, which were drawn onto coated slides and dried overnight. Each slide was manually stained with the Opal 6-Plex Manual Detection Kit - for Whole Slide Imaging (Akoya Biosciences) for CD30 (HRSC), CD68 (macrophages), CD8 (cytotoxic T-cells), FoxP3 (regulatory T-cells), LAG3 (exhaustion marker), PD1 (exhaustion marker), CD3 (T-cells) and DAPI (nucleus detection) following the manufacturer's instructions.

To remove the paraffin, the slides were incubated in 100% xylene for 20 minutes subsequently rehydrated in a descending alcohol series and washed with distilled water and PBS. The following cycle (step 1-5) was repeated four times for the first panel: CD8/Opal690, CD30/Opal570, CD68/Opal520 and FoxP3/Opal620; the second panel CD30/Opal 520, CD3/Opal 570, CD8/Opal690, PD1/Opal780, at room temperature. After each step, except after incubation of the Antibody Diluent, the slides were washed three times for 2 minutes with PBS. (1) Antigen retrieval in AR6 buffer (pH 6) or AR9 buffer (pH 9) for 45 seconds at 1000 watts and for 15 minutes at 200 watts in the microwave. Cooling down to room temperature for at least 15 minutes. (2) 2-3 drops of Antibody Diluent were applied for 10 minutes to block non-specific binding sites. (3) 150µl of the primary antibody (dilution in Antibody Diluent) incubated for 30 minutes. (4) 2-3 drops of Opal Polymer HRP were applied, containing the HRP-conjugated secondary antibody. (5) The opal fluorophore (1:100 in Amplification Diluent) was incubated for 10 minutes.

The fifth cycle (step 6-12) for LAG3/Opal780 and PD1/Opal 780 was different because Opal780 is heat labile. (6) The cycle also started with a microwave treatment and the slides then cooled down to room temperature for at least 15 minutes. (7) The Antibody Diluent incubated for 10 minutes, before (8) LAG3 (1:100 in Antibody Diluent) incubated for 30 minutes. (9) Next, the HRP-conjugated secondary antibody was applied for 10 minutes. (10) TSA-DIG (1:100 in Amplification Diluent) was then applied for 10 minutes, followed by (11) a second microwave treatment and cooldown to room temperature. The last step was (12) 1 hour incubation of Opal780 (1:50 in Antibody Diluent).

For nucleus staining Dapi (1 drop in 500µl PBS) was incubated for 5 minutes. Finally, the slides were sealed with 35µl mounting medium and the coverslips were fixed with nail polish. The slides then dried overnight at 4° Celsius before scanning. Supplementary table 3 gives information on the staining order, antibody and fluorophore concentrations and corresponding buffers.

**Scanning**

The slides were scanned with the NanoZoomer S60 (Hamamatsu).

**Digital image analysis for coexpression phenotypes**

For digital image analysis we used the open-source software QuPath v. 0.3.2 .(1) The following steps were performed on each case: (1) Image import. (2) Creation of an overlay based on the DAPI image using the *image combiner* extension. (3) Definition of the regions of interest (ROI): Three squared areas of 150 x 150 µm each were placed within a HRSC node (nTME) and minimum 75 µm distant (dTME). In dTME ROI did not include an intact B-cell follicle. (4) Cell detection using the script *StarDist*.(2) The average cell count per case was 1,230 (Standard deviation (SD) ± 255) in the nTME areas and 1,522 (SD ± 300) in the dTME areas, respectively. Cell classification was performed in eight evenly placed squared training areas of 500 x 500 µm and mean values were calculated to best cover the heterogeneity of the whole slide. In the non-auto fluorescent channels, the function "create single measurement classifier" was used; intensity thresholds for the nuclear marker FoxP3 were set at "Nucleus: mean", for CD8 and LAG3 at "Cell: mean". To avoid false positive detections in the auto fluorescent channels (520 nm, 570 nm), the "train object classifier" function was used. Here, the number of labeled objects varied between 30-50 per case. (6) The cell classifications were finally applied to the analysis areas.

**Digital image analysis for protein quantification of TARC/CCL17**

For the TARC analysis we used the same software and settings as for the TME analyses. Two squared areas of 250x250µm were placed in HRSC dense areas as ROI. The average cell count was 2500 per case (SD ± 492). For cell classification of CD30+ and TARC+ cells we used the function “train object classifier”. TARC fluorescence intensity was determined for each case per CD30+ cell area (sum of “TARC: Cell: Mean” / sum of “Cell: Area ¬µm^2”). To create comparability, we had a reference slide per run (R1-7), which was analyzed in similarly positioned ROI. Here, the TARC fluorescence intensities differed only marginally (R1=0.42, R2=0.49, R3=0.34, R4= 0.39, R5=0.36, R6=0.41, R7=0.58). A correction factor was calculated from the mean value (mean=0.43) for each case (F1=1.01, F2=0.88, F3=1.26, F4=1.11, F5=1.19, F6=1.03, F7=0.73) to minimize staining-related differences.

| **Multiplex staining** | | |  |  |  |  |  | |  | | |
| --- | --- | --- | --- | --- | --- | --- | --- | --- | --- | --- | --- |
| **Antibody** | **Company** | **Clone** | **Host** | **Clonality** | **Dilution  Antibody** | **Opal  Fluorophore** | **Dilution  Fluorophore** | | **Buffer** | | |
| CD30 | UKSH intern | BerH2 | mouse | mono | 1:10 | Opal 570 | 1:100 | | AR6 | | |
| FoxP3 | abcam | SP97 | rabbit | mono | 1:25 | Opal 620 | 1:100 | | AR6 | | |
| LAG3 | abcam | EPR20261 | rabbit | mono | 1:100 | Opal 780 | 1:50 | | AR6 | | |
| CD68 | Dako | PG-M1 | mouse | mono | 1:50 | Opal 520 | 1:100 | | AR6 | | |
| CD8 | Dako | C8/144B | mouse | mono | 1:100 | Opal 690 | 1:100 | | AR6 | | |
| CD3 | Novo Castra | LN10 | mouse | mono | 1:100 | Opal 570 | 1:100 | | AR6 | | |
| PD1 | Cell Signaling | EH33 | mouse | mono | 1:25 | Opal 780 | 1:50 | | AR6 | | |
| TIM3 | Cell Signaling | D5D5R | rabbit | mon0 | 1:25 | Opal 620 | 1:100 | | AR9 | | |
|  |  |  |  |  |  |  |  | |  | | |
| **Fluorescent staining** |  |  |  |  |  |  |  | |  | | |
| **Antibody** | **Company** | **Clone** | **Host** | **Clonality** | **Dilution  Antibody** | **Buffer** |  | |  | | |
| TARC | R&D BioTechne |  | goat | poly | 1:100 | pH9 |  | |  | | |
| CD30 | UKSH intern | BerH2 | mouse | mono | 1:10 | pH9 |  | |  | | |
| **Secondary antibodies** |  |  |  |  |  |  |  | |  | | |
| Alexa 488 | ThermoScientific |  | donkey  anti mouse |  | 1:100 |  |  | |  | | |
| Alexa 555 | ThermoScientific |  | donkey  anti goat |  | 1:100 |  |  | |  | | |
|  |  |  |  |  |  |  |  | |  | | |
| **Conventional staining** |  |  |  |  |  |  |  | |  | | |
| **Antibody** | **Company** | **Clone** | **Host** | **Clonality** | **Dilution  Antibody** | **Buffer** | | **StainiStaining Device** | |  |  |
| HLA-DP/DQ/DR | Dako | CR3/43 | mouse | mono | 1:100 | pH6 | | manual | |  |  |
| ß-2-Microglobulin | Dako |  | rabbit | poly | 1:2000 | pH6 | | manual | |  |  |
| CD30 | UKSH intern | BerH2 | mouse | mono | 1:5 | ER1 | | Bond Stainer | |  |  |
| CD20 | Dako | L26 | mouse | mono | 1:400 | ER1 | | Bond Stainer | |  |  |
| CD8 | Dako | C8/144B | mouse | mono | 1:200 | ER1 | | Bond Stainer | |  |  |

**Supplementary table 1**: Antibodies used in the study

|  | **NIVAHL Cohort** | |
| --- | --- | --- |
|  | **HLA-I positive**  **(N=11)** | **HLA-I negative**  **(n=83)** |
| **Age at enrollment – yr** |  |  |
| Median | 41 | 27 |
| Range | 19-56 | 18-60 |
| **Sex – no. (%)** |  |  |
| Female | 5 (45) | 52 (63) |
| Male | 6 (55) | 31 (37) |
| **Ann Arbor stage – no. (%)** |  |  |
| IA | 1 (9) | 2 (2) |
| IB | 0 | 1 (1) |
| IIA | 5 (45) | 65 (78) |
| IIB | 5 (45) | 15 (18) |
| IIIA | 0 | 0 |
| IIIB | 0 | 0 |
| IVA | 0 | 0 |
| IVB | 0 | 0 |
| **ECOG performance status – no. (%)** |  |  |
| 0 | 5 (45) | 68 (82) |
| 1 | 6 (55) | 15 (18) |
| 2 | 0 | 0 |
| **Risk factors – no. (%)** |  |  |
| Large mediastinal mass | 0 | 18 (22) |
| Extranodal involvement | 1 (9) | 9 (11) |
| Involvement of 3 or more nodal areas | 5 (45) | 59 (71) |
| High erythrocyte sedimentation rate | 6 (55) | 39 (47) |
| **International Prognostic Score – no. (%)** |  |  |
| 0–1 | NA | NA |
| 2–3 | NA | NA |
| 4–7 | NA | NA |
| **HLA-II – no. (%)** |  |  |
| Positive | 7 (64) | 44 (53) |
| Negative | 4 (36) | 39 (47) |

**Supplementary Table 2**: Clinical features of patient with HLA-I+ versus HLA-I- HL in the NIVAHL cohort for which molecular data were available and which are presented in this study.

| **Genes** | **p** | **95CI_low** | **95CI_high** | **HLA+_mean** | **HLA-_mean** | **Fold-Change(HLA-_to_HLA+)** | **p.adjust** |
| --- | --- | --- | --- | --- | --- | --- | --- |
| CCR4 | 4,47E-12 | -1,87654 | -1,17095 | -1,35325 | 0,170495 | 1,523745 | 3,26E-09 |
| SELPLG | 5,12E-08 | -1,14952 | -0,62297 | 0,711966 | 1,598209 | 0,886243 | 1,87E-05 |
| CD200 | 1,09E-07 | -0,98629 | -0,51141 | -0,00165 | 0,747202 | 0,748852 | 2,66E-05 |
| IL17RA | 1,51E-07 | -0,83732 | -0,43546 | 1,035268 | 1,671658 | 0,636389 | 2,71E-05 |
| PRM1 | 2,23E-07 | 0,521454 | 1,051023 | -3,96391 | -4,75014 | -0,78624 | 2,71E-05 |
| MAP2K1 | 2,48E-07 | -0,71749 | -0,3606 | 1,205011 | 1,744057 | 0,539045 | 2,71E-05 |
| RUNX1 | 2,6E-07 | -1,2392 | -0,61503 | 0,286438 | 1,213557 | 0,927118 | 2,71E-05 |
| CCL22 | 4,32E-07 | -2,90951 | -1,43164 | 0,845149 | 3,015727 | 2,170578 | 3,94E-05 |
| IL22RA1 | 5,21E-07 | 0,828958 | 1,710608 | -3,86521 | -5,13499 | -1,26978 | 4,22E-05 |
| C8A | 6,7E-07 | 0,608692 | 1,258431 | -3,81627 | -4,74983 | -0,93356 | 4,88E-05 |
| CD19 | 9,62E-07 | 0,969533 | 2,060142 | 2,135664 | 0,620827 | -1,51484 | 6,38E-05 |
| CX3CL1 | 2,13E-06 | -1,08737 | -0,48907 | -1,20174 | -0,41352 | 0,78822 | 0,000129 |
| C1R | 3,66E-06 | -1,60989 | -0,72277 | 2,991081 | 4,15741 | 1,166329 | 0,000205 |
| IL22RA2 | 4,76E-06 | -1,94655 | -0,83466 | -1,2494 | 0,141206 | 1,390603 | 0,000248 |
| HMGB1 | 5,71E-06 | -1,06448 | -0,47431 | 0,495026 | 1,264418 | 0,769392 | 0,000272 |
| DDX43 | 6,4E-06 | 0,504497 | 1,18571 | -3,36145 | -4,20655 | -0,8451 | 0,000272 |
| TMEFF2 | 6,4E-06 | 0,461231 | 1,065006 | -3,73257 | -4,49569 | -0,76312 | 0,000272 |
| PIN1 | 6,72E-06 | -0,43864 | -0,19102 | 0,886429 | 1,201259 | 0,31483 | 0,000272 |
| CAMP | 7,96E-06 | 0,450908 | 1,07752 | -4,0259 | -4,79011 | -0,76421 | 0,000305 |
| TNFSF8 | 8,91E-06 | -1,31629 | -0,55203 | -0,89117 | 0,042988 | 0,934159 | 0,000325 |
| GTF3C1 | 1,11E-05 | -0,56789 | -0,24314 | 1,216568 | 1,622081 | 0,405513 | 0,000376 |
| KLRF1 | 1,15E-05 | 0,381386 | 0,932527 | -2,55018 | -3,20713 | -0,65696 | 0,000376 |
| ANP32B | 1,19E-05 | -0,89324 | -0,38323 | 2,69536 | 3,333599 | 0,638239 | 0,000376 |
| IFNL2 | 1,53E-05 | 0,537676 | 1,297237 | -3,52511 | -4,44257 | -0,91746 | 0,000449 |
| IFNA2 | 1,56E-05 | 0,456651 | 1,105084 | -3,80179 | -4,58266 | -0,78087 | 0,000449 |
| CASP3 | 1,6E-05 | -1,08708 | -0,45154 | 0,227865 | 0,997173 | 0,769307 | 0,000449 |
| BCL10 | 1,67E-05 | -0,90838 | -0,38435 | 1,458053 | 2,104418 | 0,646365 | 0,00045 |
| CCL17 | 1,81E-05 | -3,54049 | -1,45011 | 2,77523 | 5,270528 | 2,495299 | 0,000472 |
| CD22 | 2,02E-05 | 0,627713 | 1,578867 | 3,099307 | 1,996017 | -1,10329 | 0,000501 |
| MAPK14 | 2,06E-05 | -0,32629 | -0,13072 | 2,223635 | 2,452136 | 0,228502 | 0,000501 |
| BST1 | 2,73E-05 | -1,03339 | -0,41799 | -0,72689 | -0,0012 | 0,72569 | 0,000641 |
| CCL25 | 2,95E-05 | 0,4087 | 1,047457 | -3,47394 | -4,20202 | -0,72808 | 0,000673 |
| CHUK | 4,49E-05 | 0,29611 | 0,760866 | 0,872517 | 0,344029 | -0,52849 | 0,000992 |
| RAG1 | 5,39E-05 | 0,38119 | 1,022664 | -3,3129 | -4,01482 | -0,70193 | 0,001133 |
| PTPRC | 5,57E-05 | -0,77223 | -0,29126 | 3,711824 | 4,243572 | 0,531748 | 0,001133 |
| C3 | 5,59E-05 | -1,45166 | -0,54809 | 2,557461 | 3,557339 | 0,999879 | 0,001133 |
| PSMB7 | 6,42E-05 | -0,65829 | -0,24943 | 1,651884 | 2,105746 | 0,453862 | 0,001264 |
| AIRE | 6,74E-05 | 0,499365 | 1,368154 | -2,11287 | -3,04663 | -0,93376 | 0,001283 |
| TFEB | 6,86E-05 | 0,312758 | 0,83765 | 0,80206 | 0,226856 | -0,5752 | 0,001283 |
| MAPK11 | 7,54E-05 | -0,63274 | -0,23533 | -1,6841 | -1,25007 | 0,434035 | 0,001366 |
| AICDA | 7,83E-05 | 0,449949 | 1,220626 | -2,84788 | -3,68317 | -0,83529 | 0,001366 |
| BCL2 | 7,87E-05 | 0,476527 | 1,280895 | 1,790888 | 0,912177 | -0,87871 | 0,001366 |
| IL12RB2 | 9,06E-05 | -1,31083 | -0,47323 | -1,11258 | -0,22056 | 0,892027 | 0,001536 |
| ITGA5 | 0,000105 | -1,12102 | -0,40442 | 0,861907 | 1,624629 | 0,762721 | 0,001744 |
| TIRAP | 0,000123 | 0,246627 | 0,704081 | -0,83699 | -1,31234 | -0,47535 | 0,001994 |
| IL5 | 0,000129 | 0,45074 | 1,290602 | -4,49542 | -5,36609 | -0,87067 | 0,002047 |
| CEACAM6 | 0,00014 | 0,664383 | 1,871118 | -3,41122 | -4,67897 | -1,26775 | 0,002128 |
| ROPN1 | 0,000143 | 0,392252 | 1,117915 | -3,55335 | -4,30843 | -0,75508 | 0,002128 |
| ATG7 | 0,000143 | -0,61788 | -0,21738 | 0,804991 | 1,222623 | 0,417632 | 0,002128 |
| CEACAM1 | 0,000147 | 0,362301 | 1,037871 | -1,9614 | -2,66148 | -0,70009 | 0,002137 |
| CDH5 | 0,00015 | -1,14376 | -0,3995 | -0,82532 | -0,05369 | 0,77163 | 0,002137 |
| NEFL | 0,000154 | 0,405229 | 1,19523 | -3,89435 | -4,69458 | -0,80023 | 0,002137 |
| HLA-G | 0,000155 | -1,10219 | -0,38124 | 1,75291 | 2,494627 | 0,741716 | 0,002137 |
| SH2B2 | 0,000163 | 0,229052 | 0,670133 | -0,54323 | -0,99282 | -0,44959 | 0,002197 |
| CT45A1 | 0,000185 | -2,30208 | -0,75332 | -2,24015 | -0,71245 | 1,5277 | 0,00243 |
| CRP | 0,000187 | 0,348831 | 1,047039 | -4,67657 | -5,37451 | -0,69794 | 0,00243 |
| NFKB1 | 0,00024 | -0,65941 | -0,2124 | -0,37892 | 0,056981 | 0,435906 | 0,00307 |
| TAB1 | 0,00025 | -0,65089 | -0,21703 | 0,051972 | 0,485931 | 0,433959 | 0,003147 |
| SEMG1 | 0,000257 | 0,339225 | 1,033717 | -3,70371 | -4,39019 | -0,68647 | 0,003173 |
| FEZ1 | 0,000269 | -0,65564 | -0,21226 | -0,90942 | -0,47546 | 0,433952 | 0,003264 |
| JAK2 | 0,000292 | -0,50346 | -0,16382 | 2,546578 | 2,880217 | 0,333639 | 0,003439 |
| HLA-E | 0,000292 | 0,168775 | 0,517518 | 5,444337 | 5,101191 | -0,34315 | 0,003439 |
| PSMD7 | 0,000298 | -0,31778 | -0,09985 | 2,443658 | 2,652471 | 0,208813 | 0,00344 |
| KIR3DL1 | 0,000302 | 0,265495 | 0,852456 | -3,84963 | -4,40861 | -0,55898 | 0,00344 |
| IL21R | 0,000313 | -0,93452 | -0,29886 | 1,865572 | 2,482261 | 0,616689 | 0,003514 |
| FN1 | 0,000337 | -2,14355 | -0,67727 | 2,445478 | 3,855885 | 1,410407 | 0,003726 |
| IL6R | 0,000351 | 0,232661 | 0,737045 | 0,761802 | 0,276949 | -0,48485 | 0,003818 |
| CD86 | 0,00036 | -1,06891 | -0,33241 | -0,81437 | -0,11371 | 0,700657 | 0,003854 |
| IL32 | 0,000369 | -0,88185 | -0,27873 | 3,411718 | 3,992011 | 0,580293 | 0,003894 |
| FOXP3 | 0,000425 | -1,18637 | -0,3603 | -0,05014 | 0,723189 | 0,773334 | 0,004426 |
| MPPED1 | 0,000466 | 0,288679 | 0,948414 | -3,8228 | -4,44135 | -0,61855 | 0,00478 |
| TNFRSF13B | 0,000474 | 0,430328 | 1,419525 | -0,17237 | -1,0973 | -0,92493 | 0,004803 |
| IFNG | 0,000501 | 0,432041 | 1,427599 | -1,34863 | -2,27845 | -0,92982 | 0,005008 |
| CD5 | 0,000526 | -0,88879 | -0,26349 | 1,71304 | 2,289184 | 0,576143 | 0,005181 |
| ULBP2 | 0,000548 | 0,369654 | 1,229824 | -3,52578 | -4,32552 | -0,79974 | 0,005331 |
| SH2D1B | 0,000572 | 0,260602 | 0,883917 | -2,41156 | -2,98382 | -0,57226 | 0,005488 |
| TP53 | 0,000619 | -0,52301 | -0,15448 | 1,621757 | 1,960503 | 0,338746 | 0,005859 |
| FUT7 | 0,00066 | 0,262356 | 0,902264 | -0,433 | -1,01531 | -0,58231 | 0,006158 |
| KIR_Activating_Subgroup_1 | 0,000667 | 0,425759 | 1,491295 | -5,1826 | -6,14112 | -0,95853 | 0,006158 |
| HLA-A | 0,000707 | -0,68385 | -0,19866 | 6,111918 | 6,553169 | 0,441251 | 0,006447 |
| TICAM1 | 0,000724 | 0,127626 | 0,438303 | -0,04319 | -0,32615 | -0,28296 | 0,006512 |
| MAGEA4 | 0,000796 | -1,10343 | -0,2976 | -4,26965 | -3,56914 | 0,700511 | 0,007072 |
| EPCAM | 0,000806 | 0,297966 | 1,05752 | -3,45776 | -4,1355 | -0,67774 | 0,00708 |
| CASP1 | 0,00086 | 0,216782 | 0,776125 | 0,784292 | 0,287838 | -0,49645 | 0,007461 |
| THY1 | 0,00089 | -0,96702 | -0,26947 | 2,240035 | 2,858282 | 0,618247 | 0,007571 |
| GPI | 0,000893 | -0,51642 | -0,14322 | 2,01528 | 2,3451 | 0,32982 | 0,007571 |
| CD79B | 0,000961 | 0,335504 | 1,23612 | 3,603836 | 2,818024 | -0,78581 | 0,008049 |
| KIR3DL3 | 0,00099 | 0,298987 | 1,09076 | -3,87537 | -4,57024 | -0,69487 | 0,0082 |
| MAPK8 | 0,001002 | -0,45448 | -0,12546 | -0,15692 | 0,133054 | 0,289971 | 0,008207 |
| CD160 | 0,001082 | 0,350978 | 1,301925 | -2,45348 | -3,27993 | -0,82645 | 0,008767 |
| COL3A1 | 0,001123 | -2,3513 | -0,62736 | 5,491097 | 6,980424 | 1,489327 | 0,008995 |
| USP9Y | 0,001195 | 0,691437 | 2,656876 | -1,72317 | -3,39733 | -1,67416 | 0,009466 |
| C4BPA | 0,001214 | 0,260659 | 1,00651 | -4,36476 | -4,99835 | -0,63358 | 0,009517 |
| IKBKG | 0,001365 | 0,117696 | 0,454073 | 0,755578 | 0,469694 | -0,28588 | 0,010587 |
| CD37 | 0,001434 | 0,244877 | 0,948101 | 2,222207 | 1,625718 | -0,59649 | 0,011006 |
| FCGR1A | 0,001485 | 0,291506 | 1,154989 | -1,85873 | -2,58198 | -0,72325 | 0,011273 |
| TNFRSF10B | 0,001516 | -0,3915 | -0,09815 | 0,167941 | 0,412769 | 0,244828 | 0,01139 |
| C3AR1 | 0,001564 | -1,03317 | -0,26119 | -0,21679 | 0,430384 | 0,647178 | 0,011601 |
| IFI35 | 0,001583 | -0,73192 | -0,18595 | 0,122523 | 0,581458 | 0,458935 | 0,011601 |
| IFI16 | 0,001591 | 0,089652 | 0,358268 | 3,104822 | 2,880862 | -0,22396 | 0,011601 |
| NOD1 | 0,001648 | 0,136327 | 0,550815 | -0,39108 | -0,73465 | -0,34357 | 0,011898 |
| BCL2L1 | 0,001675 | -0,40151 | -0,0999 | 2,303657 | 2,55436 | 0,250703 | 0,011972 |
| C1S | 0,001743 | -0,89895 | -0,22153 | 2,508234 | 3,068472 | 0,560237 | 0,012335 |
| IL2 | 0,001764 | 0,20465 | 0,838636 | -4,09197 | -4,61361 | -0,52164 | 0,012363 |
| PRKCD | 0,001782 | -0,52709 | -0,13063 | 1,806869 | 2,135727 | 0,328858 | 0,012371 |
| SMPD3 | 0,001859 | 0,149874 | 0,626787 | -1,90483 | -2,29316 | -0,38833 | 0,012784 |
| BCL6 | 0,001916 | -0,65158 | -0,15741 | 1,330232 | 1,734727 | 0,404495 | 0,013054 |
| IRF3 | 0,001942 | 0,149034 | 0,61156 | 0,862772 | 0,482475 | -0,3803 | 0,013106 |
| TIGIT | 0,002029 | 0,23011 | 0,96334 | 1,165356 | 0,568631 | -0,59672 | 0,013567 |
| PTGDR2 | 0,002083 | 0,18107 | 0,770955 | -3,45279 | -3,9288 | -0,47601 | 0,013806 |
| BIRC5 | 0,002149 | -0,79059 | -0,18368 | -0,41463 | 0,072504 | 0,487135 | 0,014112 |
| TLR9 | 0,002172 | 0,22618 | 0,958021 | -0,73051 | -1,32261 | -0,5921 | 0,014136 |
| TAPBP | 0,002193 | -0,69136 | -0,16334 | 3,950022 | 4,377373 | 0,427351 | 0,014146 |
| IFNA17 | 0,002233 | 0,308461 | 1,307952 | -4,17289 | -4,9811 | -0,80821 | 0,014277 |
| CYLD | 0,002274 | -0,52992 | -0,12238 | 3,017087 | 3,343236 | 0,326149 | 0,014296 |
| UBC | 0,002277 | -0,49222 | -0,115 | 6,00541 | 6,30902 | 0,303611 | 0,014296 |
| SSX1 | 0,002314 | 0,149905 | 0,64532 | -3,67203 | -4,06964 | -0,39761 | 0,014296 |
| IL9 | 0,002314 | -2,62675 | -0,59983 | -3,48416 | -1,87087 | 1,613291 | 0,014296 |
| EP300 | 0,002397 | -0,6114 | -0,14205 | 1,955783 | 2,33251 | 0,376726 | 0,014686 |
| IFNA7 | 0,002419 | 0,276718 | 1,196184 | -2,69201 | -3,42846 | -0,73645 | 0,014696 |
| MAPK1 | 0,002507 | -0,83043 | -0,19179 | 1,313868 | 1,824979 | 0,511111 | 0,014945 |
| PRAME | 0,002517 | -1,81863 | -0,4128 | -1,5035 | -0,38778 | 1,115718 | 0,014945 |
| DUSP4 | 0,00256 | -1,04193 | -0,23521 | 0,101386 | 0,739956 | 0,63857 | 0,014945 |
| CD8B | 0,002563 | 0,268815 | 1,170493 | 0,504527 | -0,21513 | -0,71965 | 0,014945 |
| FUT5 | 0,002563 | 0,231272 | 1,02137 | -4,26822 | -4,89454 | -0,62632 | 0,014945 |
| CTLA4 | 0,002741 | -0,9657 | -0,21401 | 2,349508 | 2,939363 | 0,589855 | 0,015816 |
| STAT5B | 0,002755 | -0,67883 | -0,15067 | 1,15751 | 1,572259 | 0,414749 | 0,015816 |
| ELANE | 0,002827 | 0,159141 | 0,715904 | -3,40857 | -3,84609 | -0,43752 | 0,016103 |
| CD99 | 0,002896 | -0,65889 | -0,14375 | 3,239267 | 3,640585 | 0,401318 | 0,016228 |
| CDKN1A | 0,002902 | -0,85098 | -0,18978 | 0,915332 | 1,435712 | 0,52038 | 0,016228 |
| MASP2 | 0,002916 | 0,20087 | 0,91669 | -4,36868 | -4,92746 | -0,55878 | 0,016228 |
| CCL26 | 0,002939 | -2,11162 | -0,45961 | -0,26605 | 1,019561 | 1,285614 | 0,016231 |
| MAGEA12 | 0,003029 | 0,194223 | 0,902579 | -4,38455 | -4,93295 | -0,5484 | 0,016601 |
| APP | 0,003227 | -0,61215 | -0,13046 | 2,26884 | 2,640144 | 0,371305 | 0,017549 |
| IL7R | 0,00325 | -0,80487 | -0,17209 | 3,30668 | 3,795157 | 0,488477 | 0,017549 |
| CD276 | 0,003314 | -0,97605 | -0,20807 | 0,848104 | 1,440164 | 0,59206 | 0,017765 |
| TNFRSF9 | 0,003508 | -0,81027 | -0,16795 | -0,26941 | 0,219697 | 0,489111 | 0,018664 |
| MASP1 | 0,003577 | 0,208584 | 0,99567 | -3,58194 | -4,18407 | -0,60213 | 0,018895 |
| IL25 | 0,003829 | 0,207389 | 1,006964 | -4,33849 | -4,94566 | -0,60718 | 0,019968 |
| PLAUR | 0,003835 | -1,12313 | -0,2308 | -0,87195 | -0,19498 | 0,676965 | 0,019968 |
| REPS1 | 0,004024 | -0,80089 | -0,16235 | 1,158199 | 1,63982 | 0,481621 | 0,020756 |
| IL12B | 0,004043 | 0,178415 | 0,893644 | -3,78456 | -4,32059 | -0,53603 | 0,020756 |
| ICOSLG | 0,004091 | 0,139306 | 0,704973 | -0,05703 | -0,47917 | -0,42214 | 0,020853 |
| CD81 | 0,004272 | -0,58464 | -0,11752 | 4,933616 | 5,284697 | 0,351081 | 0,021627 |
| MIF | 0,004378 | -0,44355 | -0,08762 | 2,95613 | 3,221714 | 0,265585 | 0,022011 |
| ARG1 | 0,004417 | 0,200293 | 1,024917 | -4,20519 | -4,8178 | -0,61261 | 0,022057 |
| CKLF | 0,004482 | -0,53732 | -0,10399 | 2,33823 | 2,658886 | 0,320656 | 0,022101 |
| CD244 | 0,004487 | 0,192506 | 0,978914 | -2,07159 | -2,6573 | -0,58571 | 0,022101 |
| MAGEC2 | 0,004581 | 0,204665 | 1,04506 | -4,12547 | -4,75033 | -0,62486 | 0,022415 |
| IL13 | 0,004672 | -1,66058 | -0,31939 | -1,74655 | -0,75657 | 0,989983 | 0,022707 |
| ENG | 0,004799 | -0,67077 | -0,12785 | 1,771797 | 2,171106 | 0,39931 | 0,023169 |
| PLA2G6 | 0,004856 | -0,54291 | -0,10318 | 0,133101 | 0,456147 | 0,323045 | 0,02329 |
| CCL27 | 0,00521 | 0,1743 | 0,940199 | -4,42445 | -4,9817 | -0,55725 | 0,024822 |
| MS4A1 | 0,005347 | 0,276982 | 1,520907 | 3,064434 | 2,16549 | -0,89894 | 0,025314 |
| STAT1 | 0,005472 | -0,88589 | -0,16378 | 4,022675 | 4,54751 | 0,524835 | 0,025613 |
| C9 | 0,005481 | 0,111119 | 0,603697 | -2,9345 | -3,2919 | -0,35741 | 0,025613 |
| HLA-DPA1 | 0,005538 | -0,91002 | -0,16853 | 4,358668 | 4,897942 | 0,539274 | 0,025714 |
| DEFB1 | 0,005939 | 0,168913 | 0,949714 | -4,30138 | -4,86069 | -0,55931 | 0,027404 |
| TNFRSF17 | 0,006047 | 0,167325 | 0,953192 | 0,254471 | -0,30579 | -0,56026 | 0,027504 |
| MAGEB2 | 0,006052 | -1,50765 | -0,26485 | -3,20852 | -2,32228 | 0,886248 | 0,027504 |
| MBL2 | 0,006074 | 0,249586 | 1,404189 | -3,91241 | -4,7393 | -0,82689 | 0,027504 |
| ITCH | 0,006229 | -0,44665 | -0,07927 | 1,903404 | 2,166367 | 0,262963 | 0,02801 |
| PAX5 | 0,00628 | 0,192602 | 1,104334 | 1,184867 | 0,536399 | -0,64847 | 0,02801 |
| CXCR6 | 0,006301 | -0,80986 | -0,14011 | 0,528991 | 1,003976 | 0,474985 | 0,02801 |
| NRP1 | 0,006492 | -1,11688 | -0,19347 | 1,686753 | 2,341928 | 0,655175 | 0,028685 |
| CLU | 0,006988 | 0,122395 | 0,734088 | -3,08113 | -3,50938 | -0,42824 | 0,030688 |
| IL1R1 | 0,007126 | -1,05528 | -0,17694 | 0,566842 | 1,182952 | 0,616111 | 0,031106 |
| CD53 | 0,007478 | -0,89537 | -0,14763 | 3,609282 | 4,130781 | 0,521499 | 0,032449 |
| IL6 | 0,007585 | -1,0732 | -0,17628 | -0,81268 | -0,18794 | 0,62474 | 0,03272 |
| TNFRSF12A | 0,007745 | -0,97155 | -0,15459 | -2,89058 | -2,32751 | 0,563073 | 0,033213 |
| LTA | 0,008153 | -1,03752 | -0,1633 | 1,406046 | 2,006457 | 0,600412 | 0,034757 |
| LAMP3 | 0,008211 | -1,00748 | -0,15853 | 0,266059 | 0,849066 | 0,583007 | 0,034802 |
| CFP | 0,008489 | -0,62634 | -0,09668 | 0,146432 | 0,507938 | 0,361506 | 0,035697 |
| SYCP1 | 0,008564 | 0,111142 | 0,721755 | -3,88805 | -4,3045 | -0,41645 | 0,035697 |
| CD1B | 0,008613 | -1,27817 | -0,19489 | -3,62799 | -2,89146 | 0,736529 | 0,035697 |
| TNFRSF4 | 0,008637 | -0,86022 | -0,13298 | 0,731283 | 1,227883 | 0,496599 | 0,035697 |
| SH2D1A | 0,008667 | -0,95682 | -0,1477 | 1,342709 | 1,894969 | 0,55226 | 0,035697 |
| IFITM1 | 0,008781 | -0,96699 | -0,14726 | 3,151236 | 3,708361 | 0,557125 | 0,035964 |
| IL22 | 0,008928 | 0,182327 | 1,211584 | -4,4904 | -5,18735 | -0,69696 | 0,036361 |
| IL1RAPL2 | 0,009063 | 0,109274 | 0,724253 | -3,27319 | -3,68995 | -0,41676 | 0,036401 |
| CTCFL | 0,009124 | 0,128558 | 0,854478 | -3,78981 | -4,28133 | -0,49152 | 0,036401 |
| PYCARD | 0,009134 | 0,066162 | 0,443342 | 0,502848 | 0,248096 | -0,25475 | 0,036401 |
| HLA-DOB | 0,009138 | 0,123604 | 0,828725 | 1,242334 | 0,766169 | -0,47616 | 0,036401 |
| LRP1 | 0,009254 | -1,07963 | -0,15957 | 1,574332 | 2,193932 | 0,619601 | 0,036663 |
| MYD88 | 0,009479 | 0,070764 | 0,48104 | 2,919371 | 2,643468 | -0,2759 | 0,037352 |
| CD33 | 0,009643 | 0,129884 | 0,884049 | -1,74247 | -2,24943 | -0,50697 | 0,037795 |
| SBNO2 | 0,00981 | 0,068439 | 0,467475 | 2,67809 | 2,410133 | -0,26796 | 0,038244 |
| IGF2R | 0,010189 | -0,56115 | -0,08102 | 1,018965 | 1,340054 | 0,321088 | 0,03951 |
| PDGFRB | 0,010353 | -0,88188 | -0,12432 | 1,649386 | 2,15249 | 0,503104 | 0,039932 |
| SMAD2 | 0,010561 | -0,79896 | -0,11209 | 0,689957 | 1,145484 | 0,455527 | 0,040523 |
| IFNAR2 | 0,011002 | 0,043646 | 0,317783 | 2,784379 | 2,603664 | -0,18071 | 0,041991 |
| ILF3 | 0,011122 | -0,33113 | -0,04495 | 3,120342 | 3,308384 | 0,188042 | 0,042229 |
| TNFRSF1B | 0,011234 | 0,060343 | 0,451578 | 2,112383 | 1,856423 | -0,25596 | 0,042433 |
| CD70 | 0,011449 | -0,8653 | -0,11441 | -1,14289 | -0,65303 | 0,489855 | 0,043024 |
| IL3RA | 0,01153 | -0,68769 | -0,09154 | -0,31115 | 0,078469 | 0,389615 | 0,043106 |
| SLAMF1 | 0,011673 | 0,073505 | 0,552762 | -0,1807 | -0,49383 | -0,31313 | 0,043417 |
| CFI | 0,011768 | -0,79381 | -0,10347 | -0,01198 | 0,436662 | 0,448643 | 0,043546 |
| TNFSF15 | 0,012064 | 0,09314 | 0,71272 | -1,00568 | -1,40861 | -0,40293 | 0,044417 |
| MEFV | 0,01214 | 0,133586 | 1,022403 | -2,14379 | -2,72179 | -0,57799 | 0,044474 |
| ATM | 0,012717 | 0,10329 | 0,812843 | -0,99857 | -1,45663 | -0,45807 | 0,046137 |
| TNFRSF11A | 0,012721 | -0,95049 | -0,1209 | -2,23863 | -1,70293 | 0,535694 | 0,046137 |
| CTAGE1 | 0,012859 | 0,138886 | 1,095738 | -4,21212 | -4,82943 | -0,61731 | 0,046406 |
| RORC | 0,013035 | -0,63354 | -0,07845 | -2,99607 | -2,64007 | 0,355996 | 0,046812 |
| TLR10 | 0,013151 | 0,117822 | 0,958142 | 0,757596 | 0,219614 | -0,53798 | 0,046994 |
| DPP4 | 0,013332 | 0,075718 | 0,623881 | -1,74142 | -2,09122 | -0,3498 | 0,047411 |
| CD38 | 0,013474 | -0,80418 | -0,09856 | 1,164407 | 1,615777 | 0,45137 | 0,047682 |
| IKBKE | 0,013687 | -0,43473 | -0,05243 | 2,053078 | 2,296659 | 0,243581 | 0,048202 |
| CEBPB | 0,013843 | -0,87181 | -0,10485 | 1,336492 | 1,824821 | 0,488329 | 0,048491 |
| CCR9 | 0,013902 | 0,122585 | 1,019754 | -4,21109 | -4,78226 | -0,57117 | 0,048491 |
| ECSIT | 0,014384 | 0,045647 | 0,388343 | -0,11423 | -0,33122 | -0,217 | 0,049932 |

**Supplementary table 3:** Differentially expressed genes according to HLA-I expression of HRSC in the NIVAL cohort.

| **cohort** | **HLA-I** | **HLA-II** | **EBV** | **Staining** |
| --- | --- | --- | --- | --- |
| NIVAHL | - | + | - | TARC-CD30 |
| NIVAHL | - | + | - | TARC-CD30 |
| NIVAHL | - | + | - | TARC-CD30 |
| NIVAHL | - | + | - | TARC-CD30 |
| NIVAHL | - | + | - | TARC-CD30 |
| NIVAHL | + | + | + | TARC-CD30 |
| NIVAHL | + | + | + | TARC-CD30 |
| NIVAHL | + | + | + | TARC-CD30 |
| NIVAHL | + | - | - | TARC-CD30 |
| NIVAHL | + | + | + | TARC-CD30 |
| HD14 | + | - | - | TARC-CD30; Multiplex |
| HD14 | + | + | - | TARC-CD30 |
| HD14 | + | - | - | TARC-CD30 |
| HD14 | + | - | - | TARC-CD30 |
| HD12/15 | + | - | - | TARC-CD30 |
| HD12/15 | + | - | - | TARC-CD30; Multiplex |
| HD12/15 | + | - | - | TARC-CD30; Multiplex |
| HD12/15 | + | - | - | TARC-CD30; Multiplex |
| HD12/15 | + | + | - | TARC-CD30 |
| no trial | + | - | - | TARC-CD30; Multiplex |
| no trial | - | - | + | Multiplex |
| no trail | - | - | - | Multiplex |
| no trial | - | + | - | Multiplex |
| no trial | - | + | - | Multiplex |
| no trial | - | - | - | Multiplex |
| no trial | - | + | - | Multiplex |
| no trial | - | + | - | Multiplex |
| no trial | + | - | + | Multiplex |
| HD14 | + | - | + | Multiplex |
| HD14 | + | - | + | Multiplex |
| HD12/15 | - | + | - | Multiplex |
| HD12/15 | - | - | - | Multiplex |
| HD12/15 | - | + | - | Multiplex |
| HD12/15 | - | - | - | Multiplex |
| HD12/15 | - | - | + | Multiplex |

**Supplementary table 3** Characteristics of cohort analyzed by fluorescence multi-staining. EBV=Epstein Barr Virus, + = positive, - = negative

**
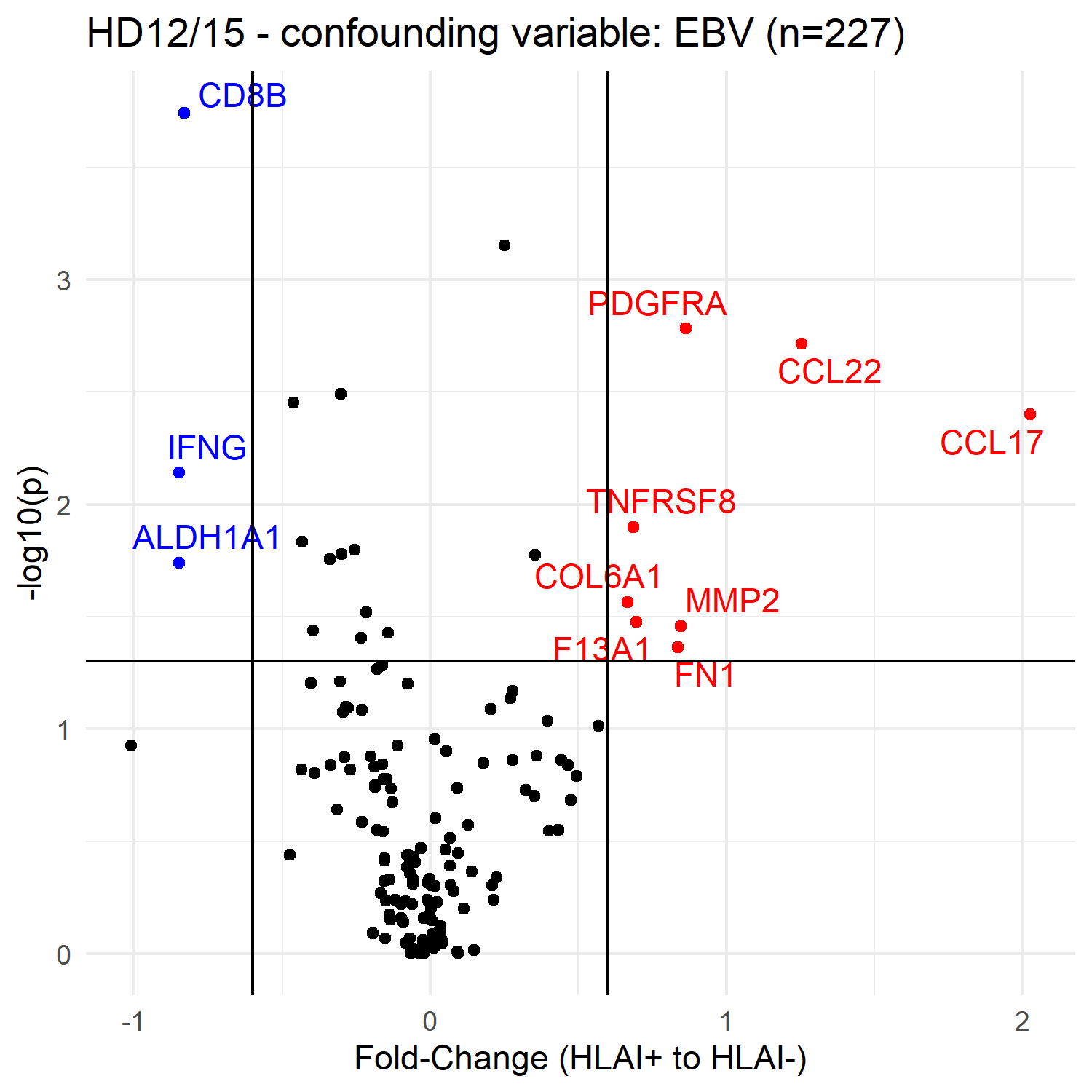
**

**Supplementary figure 1:** Volcano-plot of previously published restricted set of genes (n=142)(3) using EBV as a confounder. Blue: up-regulated in HLA-I+, Red: down-regulated in HLA-I+ HL.


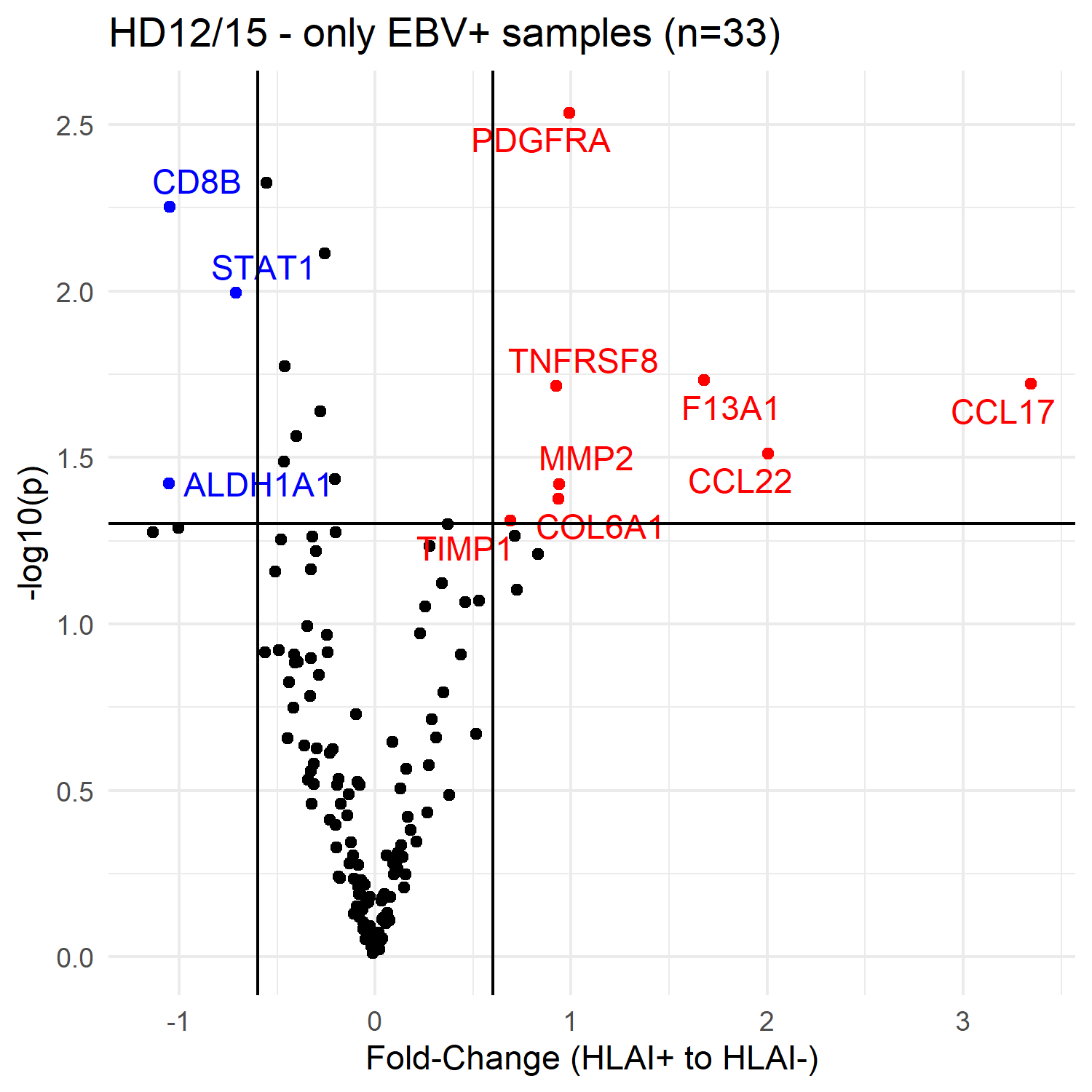


**Supplementary figure 2:** Volcano-plot of previously published restricted set of genes (n=142)(3) only in HL with EBV-positive HRSC. Blue: up-regulated in HLA-I+, Red: down-regulated in HLA-I+ HL.


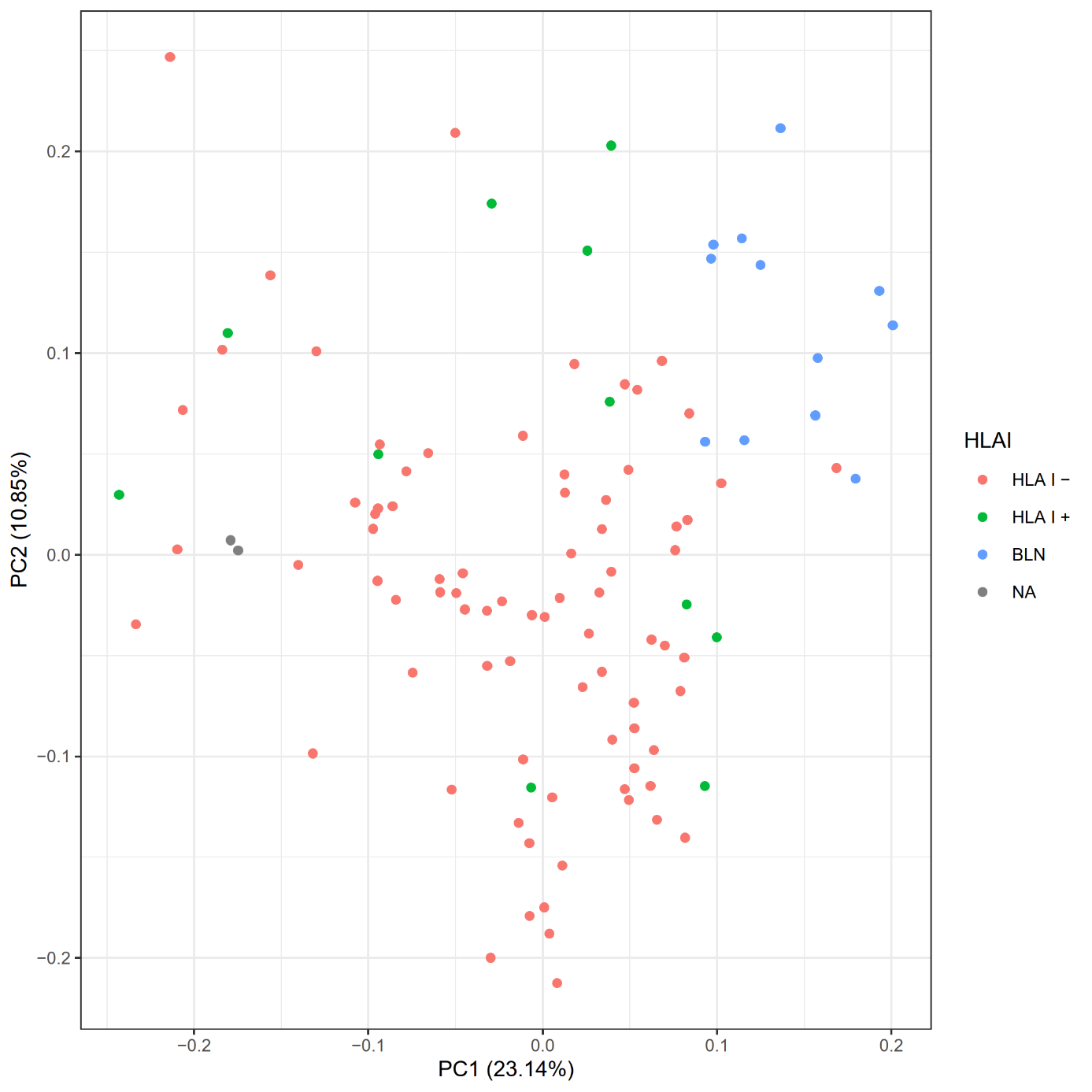


**Supplementary Figure 3** Principle component analysis of log2-transformed and mean-centered gene expressions in respect to HLA-I expression in NIVAHL cases. blue: benign lymph nodes, red: HLA-I-, green HLA-I+ treatment-naïve classic Hodgkin Lymphoma (NIVAHL cohort). grey: classical Hodgkin lymphoma not evaluable for HLA-I expression.


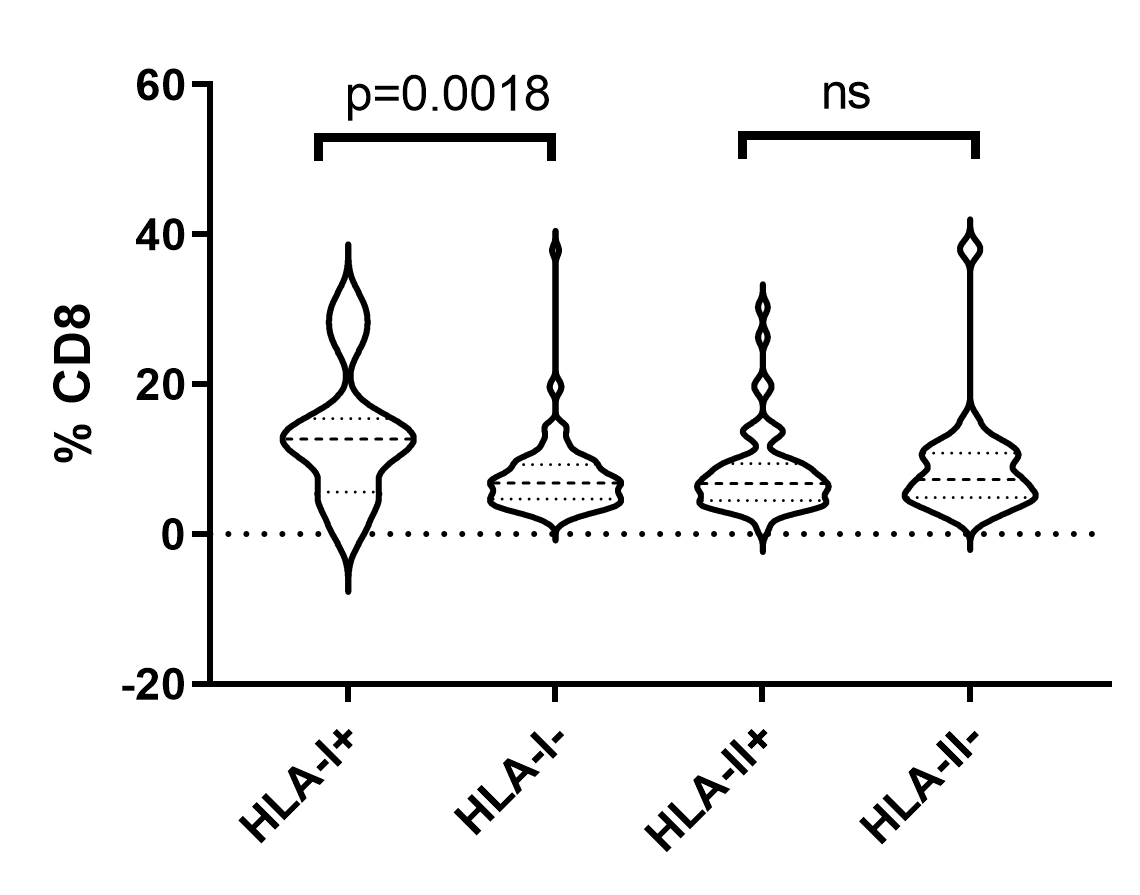


**Supplementary figure 4**: Content of CD8 cells assessed by whole slide image analysis as previously described.(4) ns= not significant.


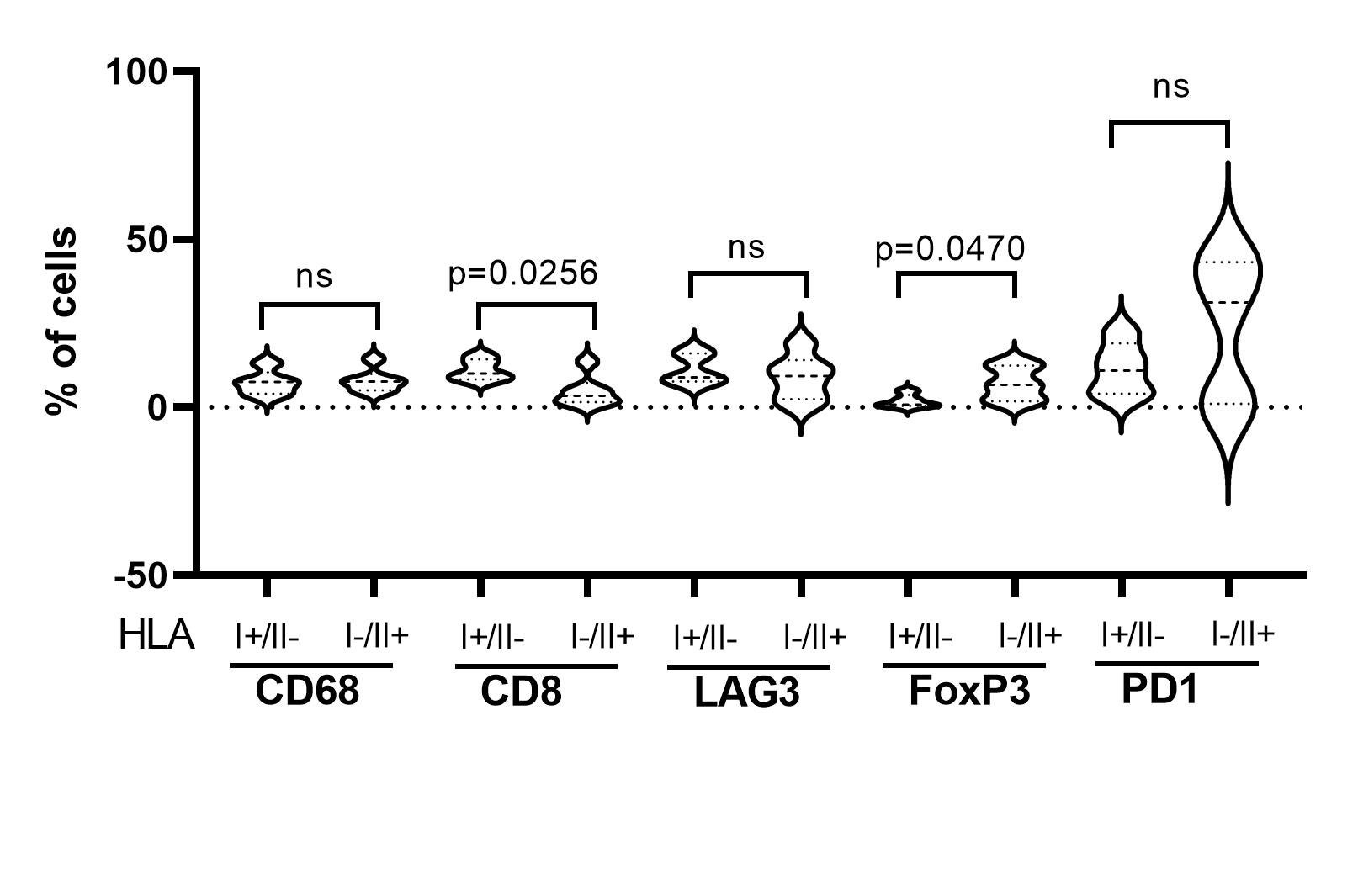


**Supplementary figure 5**: Analysis of areas in close proximity to HRSC in EBV-negative HL. Analysis restricted to cases expressing HLA-I but not HLA-II (HLA-I+) or HLA-II but not HLA-I (HLA-II+). Percent of cells in the respective areas indicated. P-values according to unpaired t-test. ns= not significant.


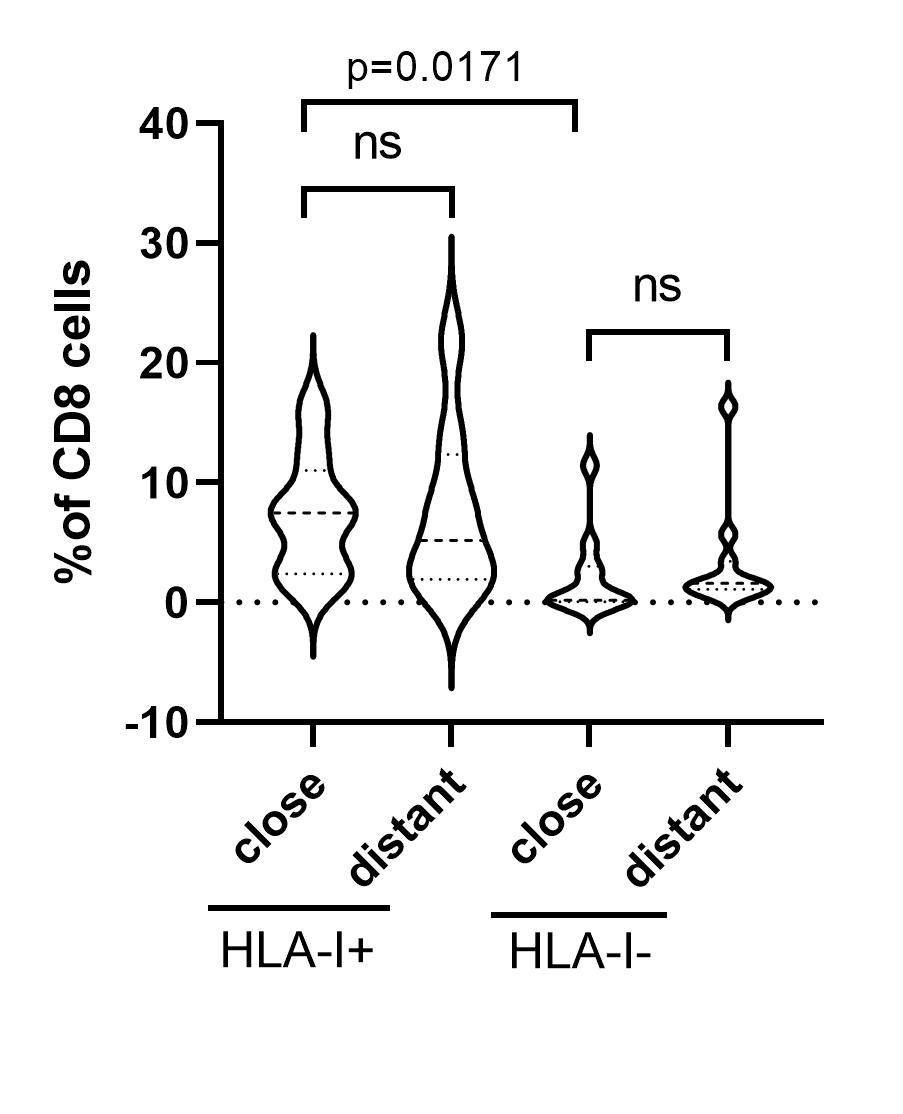


Supplementary figure 6: Expression of TIM3 in CD8+ cells according to HLA-I expression. Nn= not significant.

A)


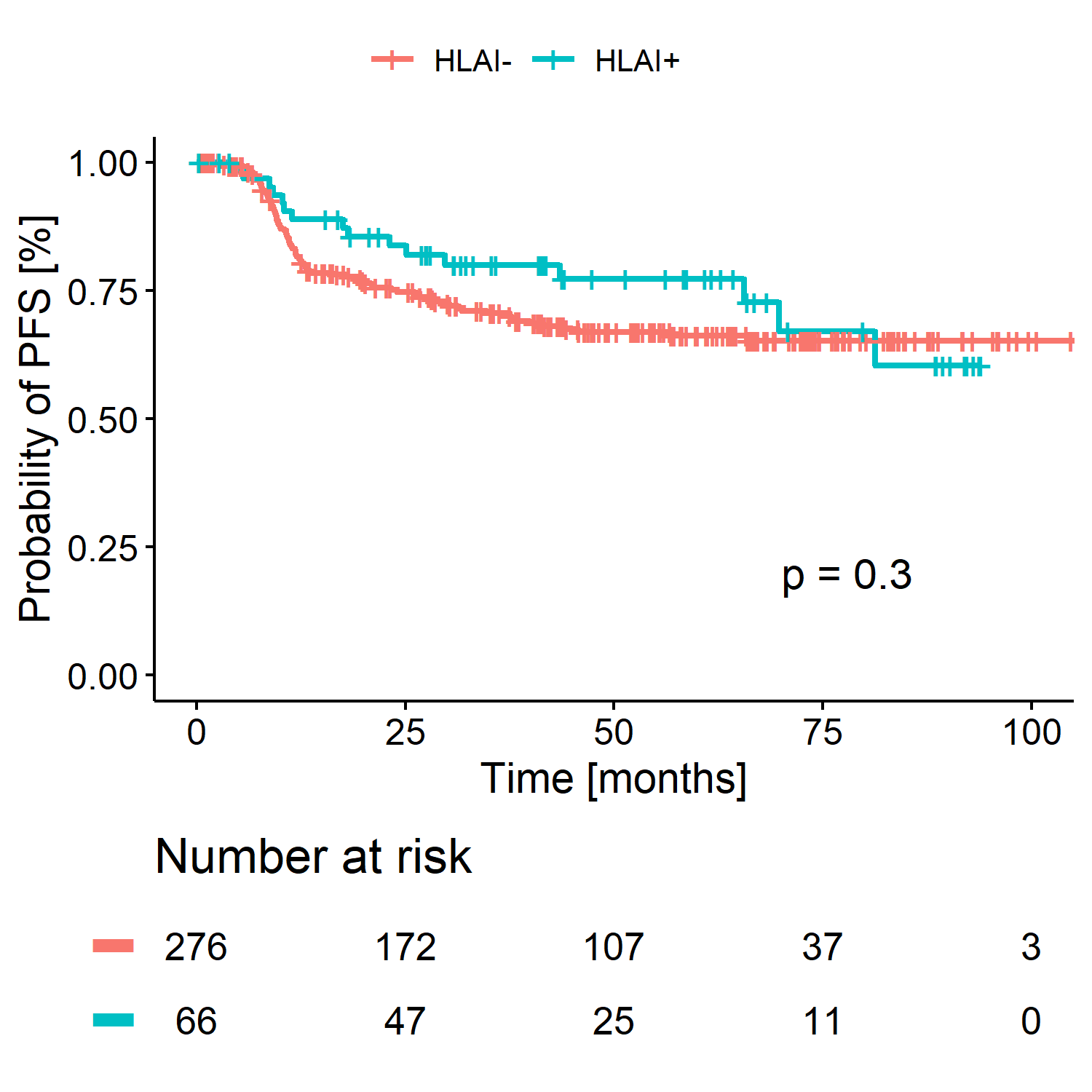


B)


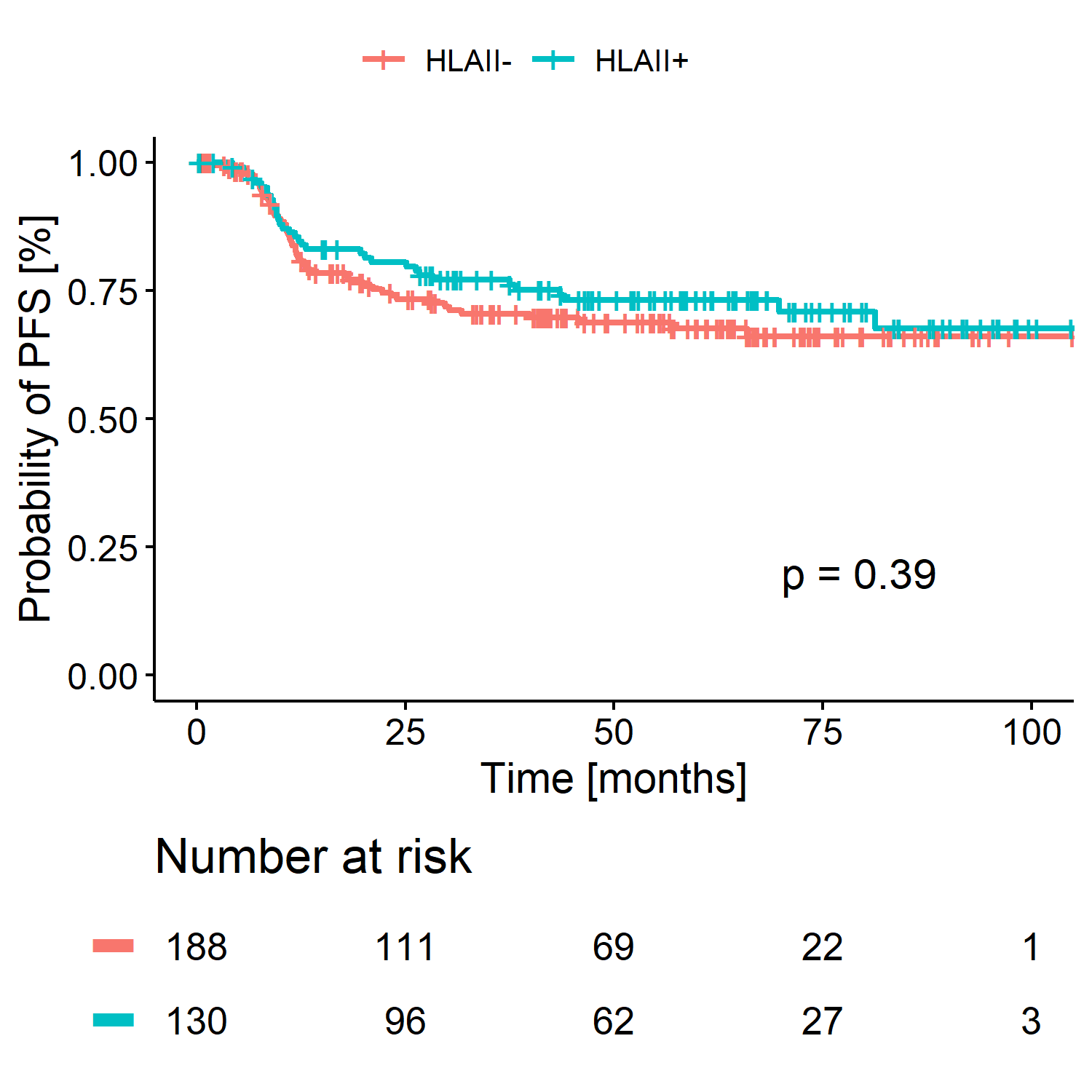


**Supplementary figure 7:** Analysis of progression free survival (PFS) of patients with advanced stage HL treated in the combined HD12/HD15 trials as previously described(3, 4) for which HLA information was available. A: patients sorted according to HLA-I and B) according to HLA-II expression.

A)


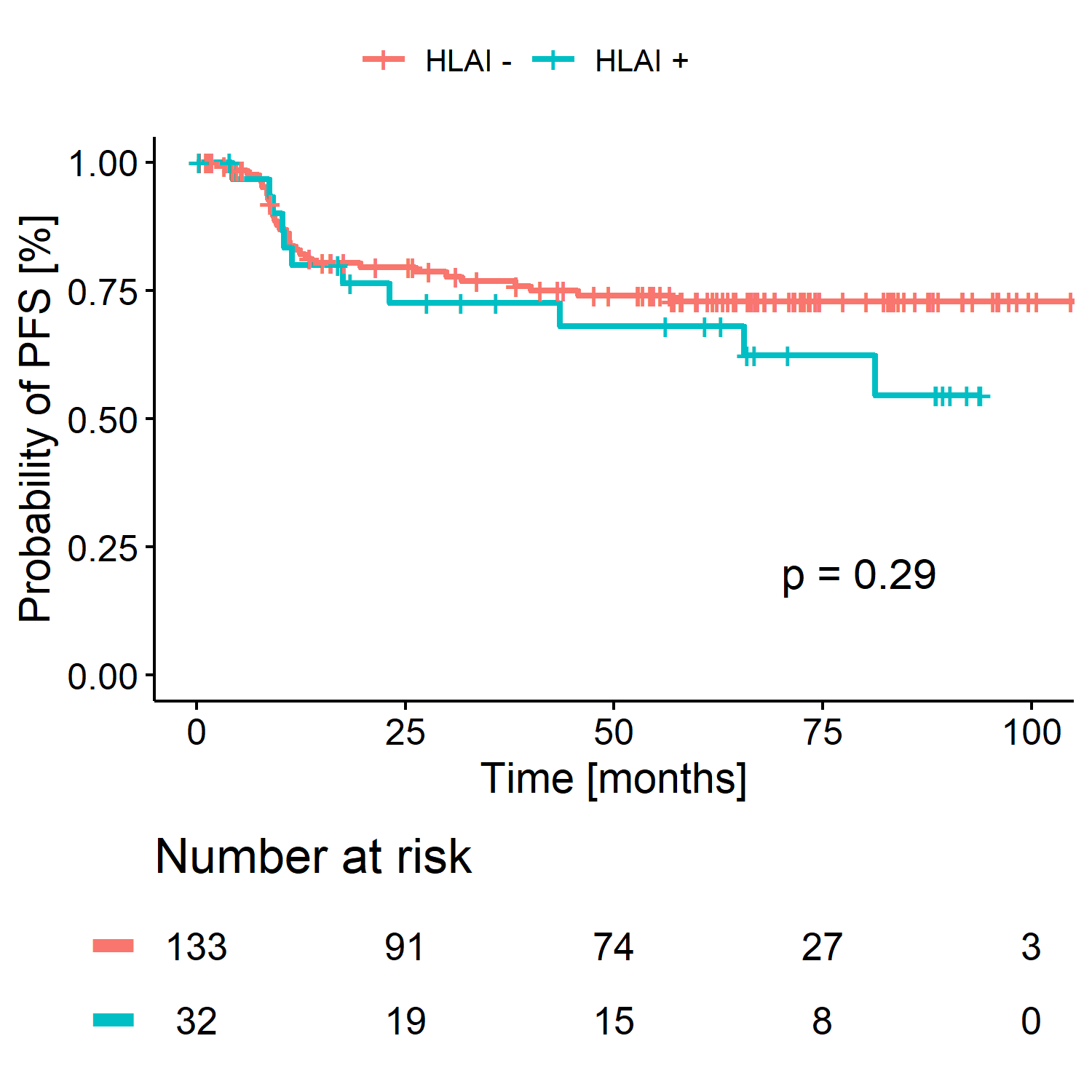


B)


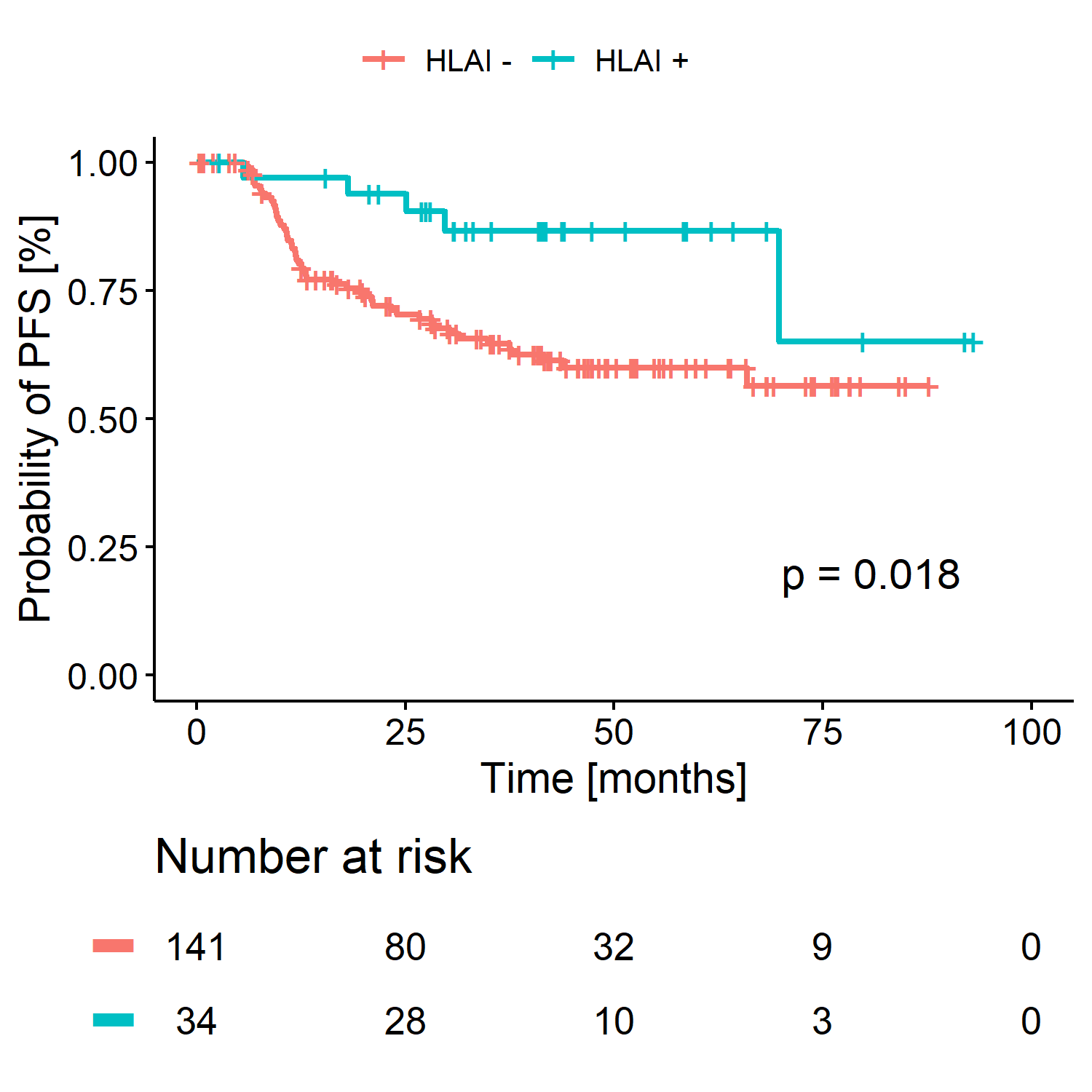


Supplementary Figure 8: Analysis of progression free survival (PFS) of patients with advanced stage HL in the HD12 (A) and HD15 (B) trial separately.

1. Bankhead P, Loughrey MB, Fernández JA, Dombrowski Y, McArt DG, Dunne PD, et al. QuPath: Open source software for digital pathology image analysis. Scientific reports. 2017;7(1):16878.

2. Schmidt U, Weigert M, Broaddus C, Myers G, editors. Cell Detection with Star-Convex Polygons2018; Cham: Springer International Publishing.

3. Jachimowicz RD, Klapper W, Glehr G, Müller H, Haverkamp H, Thorns C, et al. Gene expression-based outcome prediction in advanced stage classical Hodgkin lymphoma treated with BEACOPP. Leukemia. 2021;35(12):3589-93.

4. Jachimowicz RD, Pieper L, Reinke S, Gontarewicz A, Plütschow A, Haverkamp H, et al. Analysis of the tumor microenvironment by whole-slide image analysis identifies low B cell content as a predictor of adverse outcome in advanced-stage classical Hodgkin lymphoma treated with BEACOPP. Haematologica. 2020.
